# Rosiglitazone Induces Mitochondrial Biogenesis in Differentiated Murine 3T3-L1 and C3H/10T1/2 Adipocytes

**DOI:** 10.1155/2011/179454

**Published:** 2011-10-15

**Authors:** James X. Rong, Jean-Louis D. Klein, Yang Qiu, Mi Xie, Jennifer H. Johnson, K. Michelle Waters, Vivian Zhang, Jennifer A. Kashatus, Katja S. Remlinger, Nan Bing, Renae M. Crosby, Tymissha K. Jackson, Sam M. Witherspoon, John T. Moore, Terence E. Ryan, Sue D. Neill, Jay C. Strum

**Affiliations:** ^1^Metabolic Discovery Technology Group, Molecular Discovery Research, GlaxoSmithKline, 5 Moore Drive, Research Triangle Park, NC 27709, USA; ^2^Cheminformatics, Molecular Discovery Research, GlaxoSmithKline, 5 Moore Drive, Research Triangle Park, NC 27709, USA; ^3^Oncology Discovery Technology Group, Molecular Discovery Research, GlaxoSmithKline, 709 Swedeland Road, King of Prussia, PA 19406, USA; ^4^Discovery Analytics, GlaxoSmithKline, 5 Moore Drive, Research Triangle Park, NC 27709, USA; ^5^Integrative Biology, High Throughput Biology, Discovery Research, GlaxoSmithKline, 709 Swedeland Road, King of Prussia, PA 19406, USA

## Abstract

Growing evidence indicates that PPAR**γ** agonists, including rosiglitazone (RSG), induce adipose mitochondrial biogenesis. By systematically analyzing mitochondrial gene expression in two common murine adipocyte models, the current study aimed to further establish the direct role of RSG and capture temporal changes in gene transcription. Microarray profiling revealed that in fully differentiated 3T3-L1 and C3H/10T1/2 adipocytes treated with RSG or DMSO vehicle for 1, 2, 4, 7, 24, and 48 hrs, RSG overwhelmingly increased mitochondrial gene transcripts time dependently. The timing of the increases was consistent with the cascade of organelle biogenesis, that is, initiated by induction of transcription factor(s), followed by increases in the biosynthesis machinery, and then by increases in functional components. The transcriptional increases were further validated by increased mitochondrial staining, citrate synthase activity, and O_2_ consumption, and were found to be associated with increased adiponectin secretion. The work provided further insight on the mechanism of PPAR**γ**-induced mitochondrial biogenesis in differentiated adipocytes.

## 1. Introduction

Peroxisomal proliferators-activated receptor (PPAR*γ*) agonists, including rosiglitazone (RSG), are effective in improving insulin sensitivity in vivo. Several mechanisms have been established underlying the beneficial effects of PPAR*γ* activation, including (1) induction of adipogenesis, sequestering lipids in adipose tissues and away from the liver and muscle, (2) regulation of adipokine release, influencing the functions of other organs through regulating adiponectin and other adipokine production, and (3) modulation of inflammation, mitigating inflammatory responses in adipose tissue and other organs [1–3].

Emerging evidence supports yet another mechanism—PPAR*γ* activation also induces mitochondrial biogenesis and ameliorates the impaired mitochondrial function in adipose tissues in type 2 diabetes mellitus (T2DM) patients and rodent models, demonstrated in others' and our studies [4–7]. However, the in vivo observations could be due to an indirect effect, resulting from normalization of hyperglycemia, changes in adipose inflammation, and so forth, where PPAR*γ* activation has been shown to play a role [3]. Additionally, the ongoing adipogenesis from preadipocytes, with which mitochondrial biogenesis is associated [8], could have accounted for the increased expression of mitochondrial genes in the adipose tissue consisted of a variety of cell types. 

While a growing number of studies are supporting the direct role of PPAR*γ* activation on mitochondrial biogenesis [8–11], the cascade of changes associated with this process has not been characterized. The present study examined the temporal regulation of the transcription of virtually all known nucleus-encoded genes involved in mitochondrial function and biogenesis in differentiated murine adipocytes using gene expression profiling. Two murine cell types were used, 3T3-L1 and C3H/10T1/2 adipocytes. Both are widely accepted models for adipocyte research, but 3T3-L1 cells are considered as preadipocytes committed to adipocyte differentiation [12, 13], whereas C3H/10T1/2 are multipotent cells capable of differentiating into myoblasts and other cell types [14]. The transcriptional changes in the two adipocyte types were further validated by quantitative real-time RT-PCR (QRT-PCR), mitochondrial staining, citrate synthase activity and O_2_ consumption. The work provided unequivocal evidence for the direct role of RSG, and insight on the mechanism of adipose mitochondrial biogenesis.

## 2. Materials and Methods

### 2.1. Cell Culture and Adipocyte Differentiation

Murine 3T3-L1 preadipocytes and C3H/10T1/2 cells were purchased from American Type Culture Collection (Manassas, VA) and maintained separately in complete medium consisting of DMEM (Invitrogen, Carlsbad, CA) containing 10% fetal bovine serum (FBS; Invitrogen). All cultures were incubated at 37°C in a humidified incubator containing 5% CO_2_ and 95% air. 

For differentiation, both cell types were plated in Costar plates (Corning, Lowell, MA) precoated with 0.2% gelatin (Sigma, St. Louis, MO) in phosphate-buffered saline (PBS). 3T3-L1 preadipocytes were differentiated using a protocol similar to those described previously that included a PPAR*γ* agonist to ensure consistent differentiation [15, 16]. Briefly, cells were plated at 63,000 cells/cm^2^ and allowed to grow 3 days. The cells were then incubated with complete medium containing insulin (Invitrogen; 0.8 *μ*M), 3-isobutyl-1-methylxanthine (Sigma; 0.5 mM), dexamethasone (Sigma; 0.25 *μ*M), and RSG (GlaxoSmithKline, Research Triangle Park, NC, 100 nM) for 2 days followed by complete medium containing insulin (0.8 *μ*M) for 2 days, and complete medium alone for 3 more days.

C3H/10T1/2 cells were plated at 1.26 × 10^5^ cells/cm^2^ and allowed to grow 24 hrs in complete medium to reach confluence. Cells were then differentiated in DMEM/F12 (Invitrogen) containing 10% FBS, RSG (10 *μ*M), and insulin (1 *μ*M) for 6 days [17].

### 2.2. Adipocyte Treatments

To avoid desensitizing effects from RSG, insulin, and serum factors, prior to treatment, all differentiated murine adipocytes were incubated for 24 hrs in a washout medium, that is, DMEM/F12 medium containing 1% essential fatty acid-free bovine serum albumin (BSA, Sigma). Preliminary data indicated the 24 hr incubation did not change cell morphology or lipid content (Figure  1 of the Supplementary Material available online at doi: 10.1155/2011/179454), or decrease the mRNA levels of adipocyte markers aP2 (Fabp4) and adiponectin (Adipoq), or increase of preadipocyte/fibroblast marker platelet-derived growth factor receptor B (Pdgfrb) [18] (Tables  1(a) and 1(b) of the Supplementary Material), while optimally increasing the response to rosiglitazone using PDK4 and PCK1 as sentinel genes (Supplemental Table  1(c) of the Supplementary Material). For microarray analysis, adipocytes in 6-well plates were treated with RSG (1 *μ*M) [8] or vehicle, dimethyl sulfoxide (DMSO, Sigma) in the washout medium for 1, 2, 4, 7, 24, and 48 hrs. To ensure enough RNA yield, two wells in a plate were used for each treatment at each time point, and cells from both wells were pooled at harvest. There were 3 replicates (differentiated from 3 different batches) in each condition for each cell type.

### 2.3. Microarray Analysis

Adipocytes were lysed using QIAGEN (Qiagen Inc., Valencia, CA) lysis buffer. The cell lysate samples were transferred to QIAGEN RNeasy Mini columns mounted on a vacuum manifold. RNA isolation, DNase I treatment, reverse transcription, and cRNA synthesis, labeling, and hybridization with Genechips mouse genome 430a array containing 14,000 well-characterized mouse genes (Affymetrix Microarray Suite; Affymetrix, Santa Clara, CA) were conducted following the standard Affymetrix protocol as previously described [7]. To avoid batch effects, the RNA samples (5 *μ*g from each treatment condition) were randomized before cRNA synthesis, and the cRNA samples were rerandomized before hybridization. Array intensity data were captured by the GeneChip Computer Operating System using the algorithm (MAS 5.0; Affymetrix). All data have been deposited by MIAME standards and will be available through Gene Expression Omnibus (http://www.ncbi.nlm.nih.gov/gds; Accession number GSE14810).

### 2.4. QRT-PCR

Adipocyte RNA was subject to QRT-PCR as previously described [7]. Cidea, Lpl, Fabp4, and Cpt1b primer/probes were ordered through Applied Biosystems (Carlsbad, CA). The sequences of the rest of the genes were shown in Table  2 of the Supplementary Material, and when possible, all primer/probes were designed to span intron regions to avoid amplification of genomic DNA. The relative RNA abundance of the target genes was normalized to the housekeeping gene 36B4 (Arbp) and expressed as delta delta CT (equivalent to fold change transformed by Log_2_).

### 2.5. Oil Red O Staining

Differentiated adipocytes were washed with PBS, fixed in 10% neutral buffered formalin (Sigma), and stained (25 minutes) with Oil Red O (Sigma) saturated in isopropanol-water solution (70 : 30, v/v). Images of stained cells were immediately captured with a SPOT RT Slider camera (Sterling Heights, MI) mounted on a Leica DMIRE2 microscope.

### 2.6. Mitochondrial Staining and Quantification

Adipocytes in 96-well black Packard View Plates (PerkinElmer, Waltham, MA) were treated with RSG (1 *μ*M) or DMSO for 24 and 48 hrs and stained (45 min, 37°C) with Mito Tracker Red CMXRos (Invitrogen; 0.9 *μ*M) in the complete medium containing Hoechst (Sigma; 1 *μ*g/mL). For quantification, plates were processed on a Cellomics ArrayScan HCS Reader using a 20X objective system detecting nuclear fluorescence via a 365(50)/535(45) filter set and via a [535(35)/590(35) filter set for Mito Tracker Red fluorescence. The Cellomics “Spot Detector” BioApplication was used to derive data from images collected. The application was programmed to manage the collection of data from a minimum of 1000 cytoplasmic objects as determined by nuclear fluorescence and subsequent segmentation provided by the software. Areas of Mito Tracker Red staining were segmented by the proprietary application to effectively threshold these areas as “spots” with gating input relative to size and intensity. The resulting spot objects were analyzed to produce well-based values of “mean total spot intensity per cell” per well. The data were averaged from 8 replicate wells for each treatment and expressed as percentage of DMSO effect.

### 2.7. Citrate Synthase Activity

After treatment with RSG (1 *μ*M) or DMSO for 24 and 48 hrs, adipocytes in 24-well plates were lysed directly in CelLytic MT Cell Lysis Reagent (Sigma; 400 *μ*L), and citrate synthase activity and protein concentrations in the lysate were measured as previously described [7]. The activity was expressed as *μ*mol·min^−1^·g protein^−1^.

### 2.8. O_2_ Consumption

3T3-L1 adipocytes differentiated in 96-well plates were treated for 48 hrs with RSG at 1 *μ*M or serially diluted concentrations indicated in the Figure Legends. The cells were then washed, trypsinized, resuspended in complete medium, and transferred to 96-well BD Oxygen Biosensor System plates (BD Biosciences, Franklin Lakes, NJ). The cell suspension was sealed from air with mineral oil (Sigma) and incubated at 37°C. Consumption of O_2_ by adipocytes was associated with increased fluorescence released by the Biosensor dye coated at the bottom of the wells. At 0, 2, 4, 6, 8, 10, and 12 hrs, the raw fluorescence was measured using a fluorescent microplate reader Analyst HT (Molecular Devices, Sunnyvale, CA) with excitation and emission wavelengths of 485 and 620 nm, respectively. The data were expressed as raw fluorescent unit or percent of DMSO-treated, as indicated in Results.

### 2.9. Adiponectin Measurement

Media from 3T3-L1 adipocytes treated with RSG (1 *μ*M) or DMSO for 1, 2, 4, 7, 24, and 48 hrs were collected and diluted by 100-fold, and adiponectin concentration was determined using a Quantikine Mouse Adiponectin/Acrp30 kit (R&D Systems, Minneapolis, MN) following manufacturer's instruction.

### 2.10. Statistical Analysis

Microarray data analysis was carried out as previously described [7]. Briefly, probe sets with intensity values ≥19.9, representing the 95 percentile of the built-in Affymetrix negative control probe sets for all the chips, were considered present. Probe set intensity values were log10-transformed and analyzed using ANOVA. *P* < 0.05 and a 1.2-fold cutoff for mean fold changes were used to select genes with a significant change [7, 10]. The relative mRNA abundance from QRT-PCR, mitochondrial staining area, citrate synthase activity, measurement of O_2_ consumption, and adiponectin concentration were presented as mean ± SE in the figures. All comparisons were done using ANOVA, and *P* < 0.05 was considered statistically significant.

## 3. Results

### 3.1. Gain of Adipocyte Morphology and Increase in Differentiation Marker mRNA after Adipocyte Differentiation

**Figure 1:**
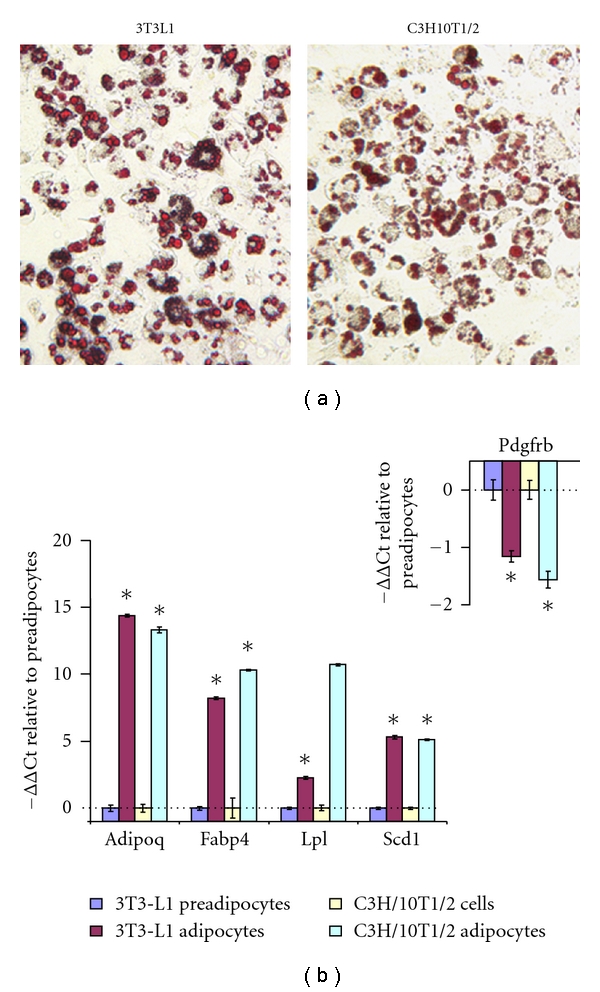
Phenotypic and molecular characterization of 3T3-L1 and C3H/10T1/2 adipocytes after differentiation. Differentiated 3T3-L1 and C3H/10T1/2 adipocytes were stained with Oil Red O ((a) Magnification = 200x). Alternatively, QRT-PCR was performed using RNA extracted from cells prior to and after differentiation (b). Relative mRNA levels of adipocyte markers Adipoq, Fabp4, Lpl, and Scd1, and preadipocyte/fibroblast marker Pdgfrb (inset in (b)) in differentiated cells were compared to those in preadipocytes. The QRT-PCR data were expressed as –ΔΔCt, equivalent to log2-transformed fold changes. All numerical data were expressed as mean ± S.E. *P < 0.05 compared to the respective preadipocytes.

At the end of differentiation, >95% of 3T3-L1 and C3H/10T1/2 adipocytes contained Oil Red O-stained lipid droplets (Figure 1(a)). The differentiation was further confirmed, using QRT-PCR, by dramatic increase in mRNA levels of Adipoq, Fabp4, lipoprotein lipase (Lpl), and stearoyl-Coenzyme A desaturase 1 (Scd1) (Figure 1(b)), and decrease in preadipocyte/fibroblast marker Pdgfrb (Figure 1(b), insets). 

Differentiated 3T3-L1 adipocytes are white adipocytes [8], and C3H/10T1/2 adipocytes have some characteristics of brown adipocytes [19], but are generally considered white adipocytes [20]. Consistent with this, we found, compared to 3T3-L1, C3H/10T1/2 adipocytes had higher levels of brown adipocyte markers uncoupling protein 1 (Ucp1) and cell death-inducing DFFA-like effector A (Cidea) mRNA, respectively (Figure  2 of the Supplementary Material). However, the levels of other brown adipocyte markers, such as type 2 deiodinase and PGC-1*α*[20] were similar between the two cell types (Table  3 of the Supplementary Material).

### 3.2. RSG Increased Gene Transcripts Encoding Proteins Regulating Mitochondrial Energy Metabolism in Differentiated 3T3-L1 and C3H/10T1/2 Adipocytes

Of the 22,626 probe sets on Affymetrix mouse chip 430a, 268 were previously [7] identified to represent genes encoding proteins localized to mitochondria and related to mitochondrial energy metabolism. These proteins include those involved in fatty acid oxidation (FAO), tricarboxylic acid cycle (TCA), oxidative phosphorylation pathway (OXPHOS), ATP/ADP shuttles, energy uncoupling proteins (UCP), and voltage-dependent anion channels (VDAC) responsible for the translocation of ATP, ADP, and other metabolites across the mitochondrial outer membrane.

**Figure 2:**
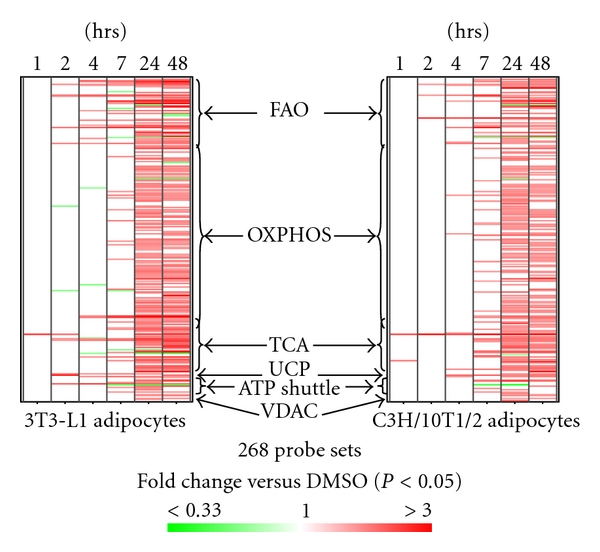
Transcriptional regulation of genes involved in mitochondrial metabolic function. Shown is a heatmap of mean fold changes of probe set intensities in 3T3-L1 and C3H/10T1/2 adipocytes treated with RSG compared to DMSO over time (*n* = 3 per time point in each cell type). Each row represents one probe set. The probe sets were grouped by their functions as indicated, and arranged in the same order for both cell types. Only fold changes significantly different (*P* < 0.05) were shown in colors (red or green).

As shown in Figure 2 and Table  4 of the Supplementary Material, in both differentiated 3T3-L1 and C3H/10T1/2 adipocytes, there was a time-dependent increase in the number of energy metabolism genes induced by RSG. At 1 hr, pyruvate dehydrogenase kinase 4 (PDK4) was the only gene in 3T3-L1 and one of the 2 in C3H/10T1/2 adipocytes significantly induced by RSG. At 2 hr, there were a few more genes induced by RSG, and among them shared by both cell types were PDK4, enoyl-Coenzyme A hydratase/3-hydroxyacyl Coenzyme A dehydrogenase (Ehhadh; an indispensable component of the FAO enzymes [21]), carnitine acetyltransferase (Crat), which modulates the acetyl-CoA/CoA ratio favoring FAO [22], and acyl-CoA synthetase long-chain family member 1 (Acsl1), a PPAR*α* target implicated in providing FA for beta-oxidation and triglyceride synthesis [23, 24]. 

The largest increase in the number of RSG-induced genes occurred at 24 hrs (70% and 63% of the 268 probe sets for 3T3-L1 and C3H/10T1/2 adipocytes, resp.), and most genes remained induced at 48 hrs (66% and 57%, resp.). Among them were those encoding key enzymes or subunits of enzymes regulating the control points [21] in the TCA cycle, namely, pyruvate dehydrogenase complex (PDC), isocitrate dehydrogenase 3, and *α*-glutarate dehydrogenase, those encoding electron transporters in OXPHOS including cytochrome c and cytochrome c1, and the enzyme controlling the rate of FAO, carnitinepalmitoyl transferase 1B (CPT1B), as well as other components of the FAO enzymes.

Ucp1 transcription was not changed in 3T3 L1 and C3H/10T1/2 adipocytes, while Cidea, controlling the protein levels and activity of AMP-activated protein kinase (AMPK) [25], was induced by RSG at 24 and/or 48 in both cell types. Both Ucp2 and Ucp3 were induced by RSG at earlier time points, that is, 2 or 4 hrs in both cell types, and remained induced through 48 hrs. 

 Few energy metabolism genes were downregulated by RSG at any time point (≤3% for both 3T3-L1 and C3H/10T1/2 adipocytes). However, among the downregulated, 3 were shared by the two adipocyte types: acyl-CoA synthetase long-chain family member 5 (Acsl5), which activates exogenous fatty acids destined for triglyceride synthesis [26], short/branched chain acyl-Coenzyme A dehydrogenase (Acadsb), and Slc25a24, which encodes a mitochondrial membrane phosphate carrier.

### 3.3. RSG Increased Gene Transcripts Encoding Proteins Regulating Mitochondrial Protein Synthesis, Transport and Folding, and Mitochondrial Structure in Differentiated 3T3-L1 and C3H/10T1/2 Adipocytes

Mitochondrial biogenesis involves both translation of mitochondrial DNA-encoded genes on mitochondrial ribosomes, and translation of nuclear DNA-encoded genes in the cytosol, followed by translocation of the nascent proteins across the mitochondrial membranes and proper folding of these proteins within mitochondria assisted by translocases of inner and outer mitochondrial membranes (TIMMs & TOMMs) and heat shock proteins (HSPs). 

**Figure 3:**
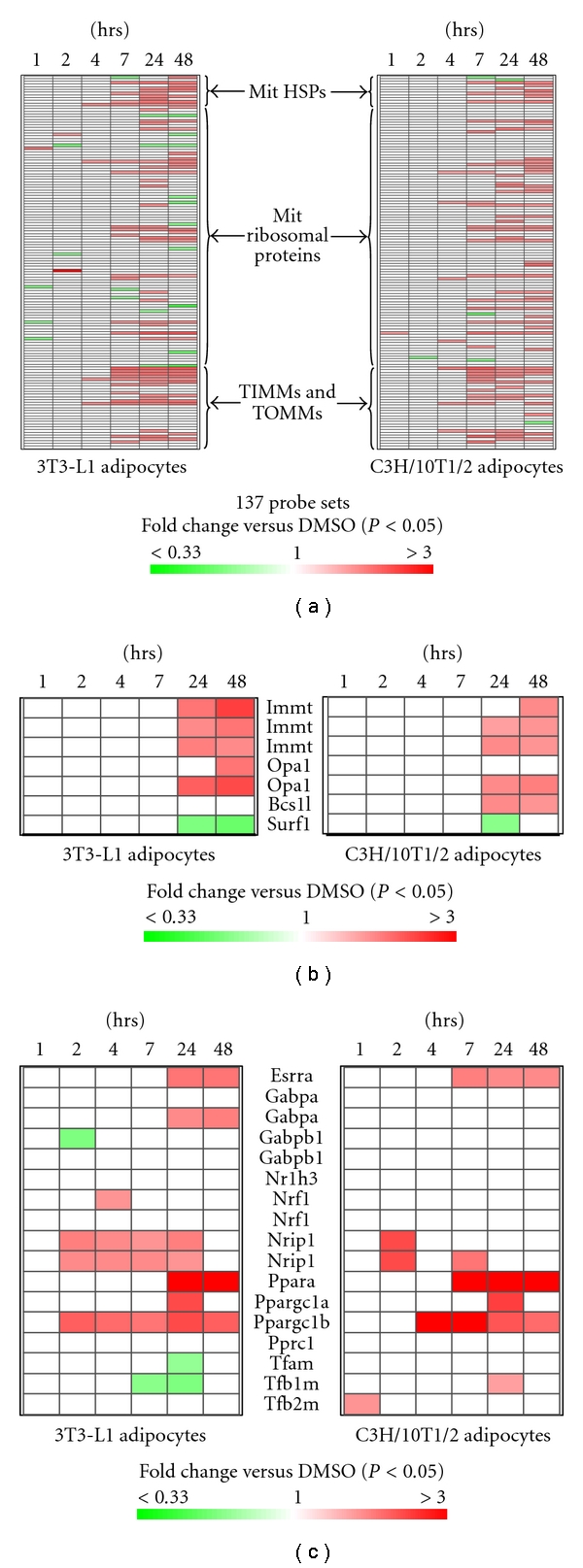
Transcriptional regulation of mitochondrial ribosomal proteins, TIMMs and TOMMs, HSPs (a), structural proteins (b), and transcription factors (c). Shown is a heatmap of mean fold changes of probe set intensities in 3T3-L1 and C3H/10T1/2 adipocytes treated with RSG compared to DMSO over time (*n* = 3 per time point in each cell type). Each row represents one probe set. Certain genes were represented by more than one probe set as indicated. Only fold changes significantly different (*P* < 0.05) were shown in colors (red or green).

As shown in Figure 3(a), of the 137 probe sets representing mitochondrial HSPs, ribosomal proteins, and TIMMs & TOMMs, few were affected by RSG at 1, 2, and 4 hrs. However, at 7 hrs after RSG treatment, 18% and 23% of the genes were induced in 3T3-L1 and C3H/10T1/2 adipocytes, respectively, whereas the rest remained unchanged except for a few (2%) that were downregulated. The number of induced genes increased slightly at 24 and 48 hrs in both cell types.

RSG also induced mitochondrial structural proteins not directly involved in metabolic function (Figure 3(b)), including mitofilin (Immt), optic atrophy 1 (Opa1) at 24 and 48 hrs in both 3T3-L1 and C3H/10T1/2adipocytes, and BCS1-like protein (Bcs1l) in C3H/10T1/2 at 24 and 48 hr, but unaffected in 3T3-L1 adipocytes. Surfeit 1 (Surf1), previously found to be induced by RSG in db/db adipose tissue but not in DIO mice [7], was downregulated by RSG in 3T3-L1 and C3H/101/2 adipocytes at 24 and/or 48 hrs.

### 3.4. RSG Effects on Transcription Factors Regulating Mitochondrial Biogenesis

Ligand-bound PPAR*γ* could regulate the expression of mitochondrial genes by interacting with transcription factors that act directly at the promoters of these genes. Alternatively, PPAR*γ* activation could induce or suppress the expression of nuclear factors that in turn interact with mitochondrial gene promoters. To evaluate the latter possibility, RSG effects on the transcription of transcription factors/cofactors known to regulate mitochondrial gene expression [27–30] were examined. As shown in Figure 3(c), PGC-1*β* (Ppargc1b) was induced at 2 and 4 hr in 3T3-L1 and C3H/10T1/2 adipocytes, respectively, and the induction remained through 48 hrs. Meanwhile, PGC-1*α* (Ppargc1a) was induced by RSG only at 24 hr in both cell types. PPAR*α* (Ppara) and ERR*α* (Essra) were both induced by RSG at 24 and 48 hr in 3T3-L1 adipocytes, and 7, 24, and 48 hr in C3H/10T1/2 adipocytes. Surprisingly, the transcriptional corepressor RIP140 (Nrip1) was induced by RSG in both cell types at 2 hrs, and remained induced through 24 hrs in 3T3-L1 adipocytes, but returned to the level comparable to that in the DMSO-treated after 2 hrs in C3H/10T1/2 adipocytes. There was no effect, or no consistent effect between the two cell types, on other transcription factors/cofactors, including nuclear respiratory factor 1 (Nrf1) and 2 (Gabpa and Gabpb1), PPAR*γ* cofactor-related 1 (Pprc1), mitochondrial transcription factor A (Tfam), B1 (Tfb1m), B2 (Tfb2m), and LXR*α* (Nr1h3), shown recently to be capable of inhibiting the brown adipose phenotype [29].

### 3.5. Validation of Microarray Findings

The transcription of 9 representative genes encoding control point enzymes or their subunits, including Cpt1b (FAO), Idh3a (TCA cycle), and Cycs (OXPHOS), as well as citrate synthase (Cs; TCA cycle), hydroxyacyl-Coenzyme A dehydrogenase/3-ketoacyl-Coenzyme A thiolase/enoyl-Coenzyme A hydratase, alpha subunit (Hadha; FAO), Opa1 (structural protein), Cidea, Ppargc1b, and Ucp1 were examined by QRT-PCR using RNA from 3T3-L1 and C3H/10T1/2 adipocytes. The results generally agreed with the microarray data (Table  5 of the Supplementary Material) except that the increase of Ppargc1b mRNA by RSG in C3H/10T1/2 adipocytes appeared to be statistically significant at 2 hr by QRT-PCR but not by microarray.

**Figure 4:**
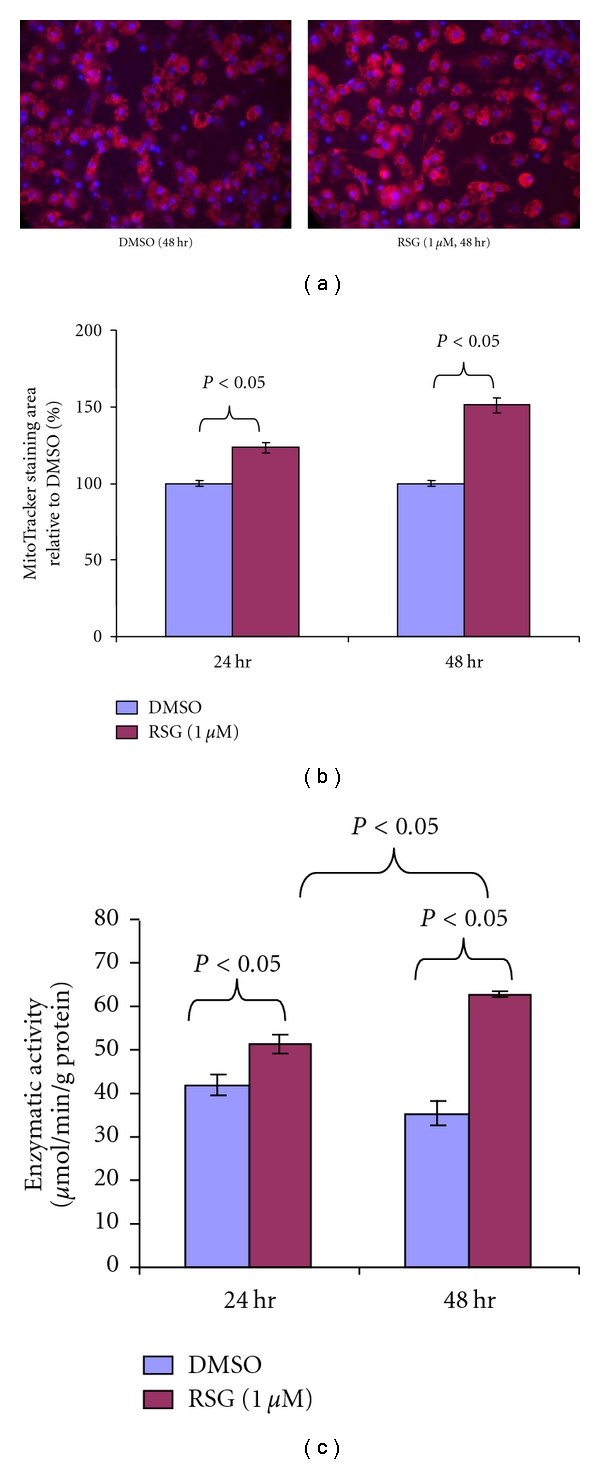
RSG increased mitochondrial staining and citrate synthase activity in 3T3-L1 adipocytes. (a) Representative pictures of 3T3-L1 adipocytes stained with Mito Tracker Red CMXRos (for mitochondria, red) and Hoechst (for nucleus, blue). Magnification = 400x. (b) Quantification of mitochondrial staining (*n* = 8 each treatment). (c) Measurement of citrate synthase activity (*n* = 2 each condition). All numerical data were expressed as mean ± S.E. All experiments were repeated at least once.

To investigate whether the increased mitochondrial gene transcription was associated with increased mitochondrial mass, we conducted live mitochondrial staining using Mito Tracker Red CMXRos in 3T3-L1 adipocytes. RSG increased mitochondrial staining area to 123% and 151% of the DMSO control at 24 and 48 hrs, respectively (Figures 4(a) and 4(b)).

To confirm the increased mitochondrial staining was associated with increased mitochondrial metabolic functions, we examined RSG effects on citrate synthase activity and O_2_ consumption. RSG significantly increased citrate synthase activity at 24 hrs, and continued to increase the activity, reaching 177% of the DMSO control at 48 hrs (Figure 4(c)). 

**Figure 5:**
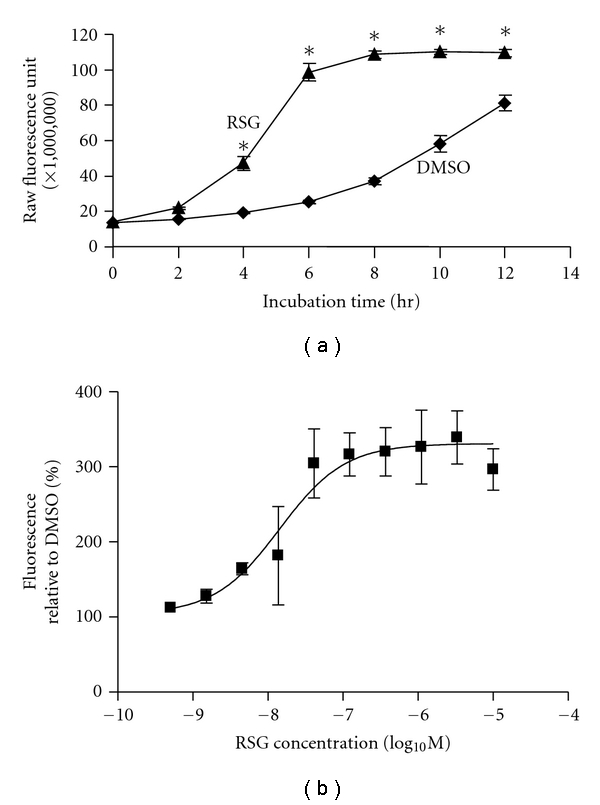
RSG increased O_2_ consumption rate in 3T3-L1 adipocytes. (a) After treatment with RSG (1 *μ*M) or DMSO (*n* = 8 each) for 48 hrs, 3T3-L1 adipocytes were transferred to the Oxygen Biosensor plates, and assayed for O_2_ consumption at 0, 2, 4, 6, 8, 10, and 12 hrs after the transfer (see Methods for more details). *P < 0.05 versus DMSO at matching time points. (b) After treatment with RSG (10, 3.3, 1.1, 0.37, 0.12, 0.041, 0.014, 0.0046, 0.0015, and 0.00051 *μ*M) or DMSO (*n* = 3 each condition) for 48 hrs, 3T3-L1 adipocytes were transferred to the Oxygen Biosensor plates and assayed for O_2_ consumption 6 hrs after the transfer. All numerical data were expressed as mean ± S.E. All experiments were repeated at least once.

To examine RSG effects on cell respiratory rate, 48 hrs after treatment, 3T3-L1 adipocytes were transferred to O_2_ Biosensor plates free of RSG, and the decrease of O_2_ concentrations (leading to increased fluorescence) in the cell suspension was monitored. The difference in O_2_ consumption between RSG- and DMSO-treated adipocytes increased with time, reaching the maximum (3~4-folds) 6 hrs after the transfer (Figure 5(a)). In a separate experiment, 3T3-L1 adipocytes were treated with increasing concentrations of RSG for 48 hrs, followed by incubation in an O_2_ biosensor plate for 6 hrs. The RSG-induced O_2_ consumption was dose dependent (Figure 5(b)).

### 3.6. Association of Adiponectin Secretion with Adipose Mitochondrial Biogenesis

**Figure 6:**
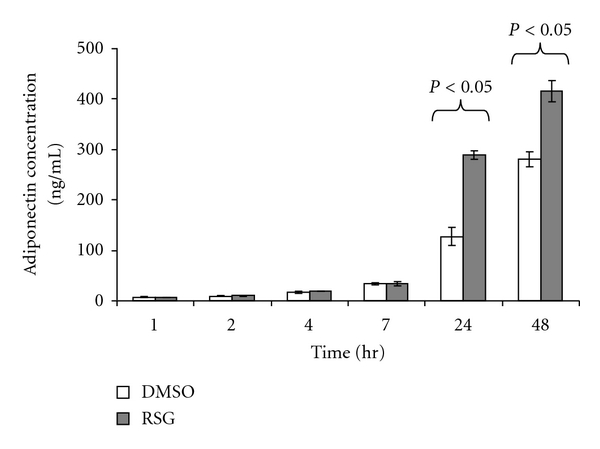
RSG induced adiponectin release from 3T3-L1 adipocytes. Cell culture medium was collected at indicated time points, and adiponectin concentration was measured (*n* = 4 each condition at each time point). All numerical data were expressed as mean ± SE. The experiment was repeated at least once.

Adipose mitochondria were recently shown to be essential in adiponectin production [31]. We therefore examined the association of adiponectin secretion and RSG-induced mitochondrial biogenesis in differentiated 3T3-L1 adipocytes. Adiponectin levels increased with time in the medium from both RSG- and DMSO-treated adipocytes (Figure 6); however, there was significantly more adiponectin production from the RSG-treated adipocytes at 24 and 48 hrs compared to the DMSO-treated adipocytes.

## 4. Discussion

In the current study, the effects of RSG on mitochondrial gene expression in 3T3-L1 and C3H/10T1/2 adipocytes were examined in a systematic manner. RSG induced the transcription of genes encoding proteins regulating mitochondrial energy metabolism, proteins responsible for mitochondrial protein synthesis, transport and folding, and mitochondrial transcription factors in both cell types. The timing of the changes was consistent with the cascade of mitochondrial biogenesis, that is, initiated by upregulation of transcription factor(s) such as PGC-1*β* (starting at 2 hours of RSG treatment), followed by increase in the biosynthesis machinery (7 hours) and then by increase in the functional components of the organelle (24 hours). Furthermore, the transcriptional increases by RSG were associated with increased mitochondrial staining, citrate synthase activity, and O_2_ consumption. Our results validated a direct role of RSG in the stimulation of adipose mitochondrial biogenesis and function.

The two murine cell types examined were derived from embryos of different mouse strains [32, 33], capable of committing to different cell lineages [14], and were differentiated to adipocytes with distinct protocols. While some adipocyte-related genes, such as adipsin (Adn), fatty acid synthase (Fasn), glucose transporter 1 (Slc2a1), responded to RSG in a manner considerably different between the two cell types (Figure  3 of the Supplementary Material), a substantial list of mitochondrial genes was affected by RSG in a strikingly similar manner. The results were consistent with previous findings in murine [8, 10] and human adipocyte cultures [9] as well as in adipose tissues [4–7, 34], indicating the effect of PPAR*γ* activation on mitochondrial biogenesis is prevalent among adipocytes derived from different conditions.

It is of interest that among mitochondrial genes PDK4 was the first responding to RSG treatment transcriptionally (Table  4 of the Supplementary Material). PDK4 inactivates PDC, which catalyzes the oxidative decarboxylation of pyruvate and thus links glycolysis to the energetic and anabolic functions of the TCA cycle [21]. While PPAR*γ* activation induced PDK4 mRNA in 3T3-L1 adipocytes and rat adipose tissues [34, 35], the implication of PDK4 induction in the tissue has not been substantially explored. In the heart, liver, and skeletal muscle, PDK4 expression increases with starvation and high-fat feeding when free fatty acids become the major energy source, and PDK4 plays an important role facilitating FAO, using fatty acids for energy production [36]. In the current study, increased PDK4 transcription by RSG in adipocytes might, like in the aforementioned tissues, facilitate catabolizing fatty acids, although cells' access to glucose in the medium was not limited. Consistent with this, proteins facilitating FAO, including Ehhadh, Crat, and Acsl1 (Table  4 of the Supplementary Material), were among the few that were induced as early as 2 hr, along with most of the other FAO genes that followed at later time points. 

The induction of FAO genes (Figure 2), associated with increased FAO capacity confirmed by increased capability to consume O_2_ (Figure 5), took place when the two adipocyte types had been incubated for more than 24 hrs in the washout medium free of serum and supplementation of fatty acids. The free fatty acid substrate for oxidation may have derived from hydrolysis of triglycerides stored in the adipocytes, because transcription of genes regulating lipolysis, for example, hormone sensitive lipase (Lipe) and adipose tissue triglyceride lipase (Pnpla2), was induced by RSG (Table  4 of the Supplementary Material). Consistent with this, RSG has been previously shown to induce lipase transcription and lipolysis in adipocytes and adipose explants [10, 37].

The increased PGC-1*β*, PGC-1*α*, ERR*α*, and PPAR*α* transcription in 3T3-L1 and C3H/10T1/2 adipocytes was consistent with previous in vivo findings that RSG induced some [8, 9] or all [7] of these transcription factors in adipose tissues from DIO, db/db, ob/ob mice, and T2DM patients. PGC-1*β* was one of the earliest induced by RSG in both adipocyte types, and the induction lasted through 48 hrs (Figure 3(c)). PGC-1*β* overexpression in muscle was associated with increased mitochondrial biogenesis, O_2_ consumption, and expression of FAO genes and OXPHOS electron transporters [38, 39]. While PGC-1*β* is thought to be less frequently regulated at the transcriptional level compared to PGC-1*α* [40], with which PGC-1*β* shares a complementary role [41], Powelka et al. showed RIP140 knockdown in 3T3-L1 adipocytes augmented mitochondrial biogenesis associated with increased PGC-1*β* transcription without changing PGC-1*α* mRNA level [27]. The current result indicates PGC-1*β* may be an immediate target of PPAR*γ* activation and may play a pivotal role in RSG-induced mitochondrial biogenesis.

It is surprising that RIP140, a negative regulator of mitochondrial biogenesis [27, 42] and antagonist of PGC-1*α* [43], was also among the earliest transcription factors/cofactors induced by RSG (Figure 3(c)). RIP140 is known to interact with PPAR*γ* [44], and RIP140 transcription increased during adipocyte differentiation from preadipocytes [27, 45], a process associated with marked mitochondrial biogenesis [8]. The relationship between RIP140 transcription and RSG-induced adipose mitochondrial biogenesis is worth further investigation. 

The induced transcription of a vast number of mitochondrial genes by RSG suggests there is general presence of PPAR-responsive elements (PPREs) in the promoter regions of these genes. In fact, confirmed PPREs have been documented for quite a few genes, including some of the early responsive ones, PDK4, Acsl1, and Ucp3, as well as others such as Cbt1b, Timm13a, PPAR*α*, and so forth [24, 46–48]. In silico analysis revealed 143 of the mitochondrial genes contained at least one exact match with PPRE core sequence [24] in the promoter regions between +100 and −2000 base-pairs relative to the transcription start site (Table  6 of the Supplementary Material). Among the genes, in addition to most of those mentioned above, were PGC-1*β*, ERR*α* and a number of those involved in energy metabolism and mitochondrial biogenesis. While the role of the PPREs needs further validation via other experimental approaches, their prevalence is consistent with the implication of a direct role of PPAR*γ* in inducing adipose mitochondrial biogenesis.

It is intriguing to speculate the physiological relevance of adipose mitochondrial biogenesis. Kaaman et al. [49] recently showed there was strong association between mitochondrial DNA copy number and lipogenesis in human white adipose tissue from obese but otherwise healthy human volunteers. Lipogenesis needs ATP, and thus mitochondria, and one way by which adipose tissue participates in whole-body energy homeostasis is through lipid storage. Another way that links the tissue to energy metabolism is through its endocrine function, that is, secretion of adipokines, particularly adiponectin, also known as the most abundant secretory protein from adipocytes [50, 51]. A recent study showed ATP generation by mitochondria is essential to adiponectin production in T2DM mice and adipocyte cultures [31]. Consistent with this, we found RSG induced adiponectin secretion (Figure 6). 

In conclusion, a direct, stimulatory role of RSG on mitochondrial biogenesis was demonstrated in two common murine adipocyte models. The time course of the transcriptional regulation was mapped. PDK4 was the first induced gene, followed by a number of genes facilitating FAO/TCA/OXPHOS, leading to increased FAO capacity. PGC-1*β* was among the first responsive transcription factors, implying its potentially pivotal role. The effect of RSG on mitochondrial function was linked to fatty acid metabolism and adiponectin production in differentiated adipocytes, revealing an underexplored mechanism by which PPAR*γ* improves insulin sensitivity in vivo. 

## Supplementary Material

Figure 1. Triglyceride content in differentiated C3H/10T1/2 adipocytes incubated in washout buffer.
Figure 2. Relative mRNA abundance of brown adipose tissue markers Ucp1 and Cidea in C3H/10T1/2 adipocytes and 3T3-L1 adipocytes, determined by QRT-PCR.
Figure 3. Adipsin (Adn), fatty acid synthase (Fasn), and glucose transporter 1 (Slc2a1), were regulated differently by RSG in 3T3-L1 and C3H/10T1/2 adipocytes.
Table 1. Regulation of adipocyte and fibroblast/preadipocyte marker genes with the washout conditions in C3H/10T1/2 cells (A), and 3T3 L1 cells (B), and responses of PDK4 and PCK1 to rosiglitazone in C3H/10T1/2 and 3T3-L1 adipocytes (C).
Table 2. QRT-PCR primer/probe sequences.
Table 3. Expression of brown adipose tissue markers in 3T3 L1 and C3H/10T1/2 adipocytes.
Table 4. Regulation of genes by RSG in 3T3-L1 (A) and C3H/10T1/2 (B) adipocytes over time.
Table 5. Confirmation of the microarray data using QRT-PCR.
Table 6. Presence of PPAR-responsive element consensus regions on the promoters of nucleus-encoded mitochondrial genes.
