# Supplementary material for: Rosiglitazone Induces Mitochondrial Biogenesis in Differentiated Murine 3T3-L1 and C3H/10T1/2 Adipocytes
Source: PPAR Res. 2011 Oct 15;2011:179454. doi: 10.1155/2011/179454 (PMC3195302; doi:10.1155/2011/179454)
Supplement: Supplementary file 1 — Figure 1. Triglyceride content in differentiated C3H/10T1/2 adipocytes incubated in washout buffer. Figure 2. Relative mRNA abundance of brown adipose tissue markers Ucp1 and Cidea in C3H/10T1/2 adipocytes and 3T3-L1 adipocytes, determined by QRT-PCR. Figure 3. Adipsin (Adn), fatty acid synthase (Fasn), and glucose transporter 1 (Slc2a1), were regulated differently by RSG in 3T3-L1 and C3H/10T1/2 adipocytes. Table 1. Regulation of adipocyte and fibroblast/preadipocyte marker genes with the washout conditions in C3H/10T1/2 cells (A), and 3T3 L1 cells (B), and responses of PDK4 and PCK1 to rosiglitazone in C3H/10T1/2 and 3T3-L1 adipocytes (C). Table 2. QRT-PCR primer/probe sequences. Table 3. Expression of brown adipose tissue markers in 3T3 L1 and C3H/10T1/2 adipocytes. Table 4. Regulation of genes by RSG in 3T3-L1 (A) and C3H/10T1/2 (B) adipocytes over time. Table 5. Confirmation of the microarray data using QRT-PCR. Table 6. Presence of PPAR-responsive element consensus regions on the promoters of nucleus-encoded mitochondrial genes. [file 179454.f1.pdf]

Supplemental Materials - Figures & Legends

Supplemental Figure 1. Triglyceride content in differentiated C3H/10T1/2 adipocytes incubated in washout buffer. After differentiation, C3H/10T1/2 adipocytes were incubated in the washout medium for 0, 1, 2, or 3 days. The medium was then removed, and adipocytes were permeated with digitonin. Total cellular triglycerides were then hydrolyzed by a triglyceride lipase into glycerol and free fatty acids. Total glycerol was then measured by coupled enzymatic reactions involving glycerol kinase, glycerol phosphate oxidase and peroxidise, leading to production of a quinoneimine dye that shows an absorbance maximum at 540 nm. According to manufacturer's instruction (Sigma), the increase in absorbance at 540 nm is directly proportional to triglyceride concentration in the cells, and therefore, cellular total triglyceride content was expressed as optical density (absorbance) at 540 nm. * $P < 0.05$ vs 0 day washout, ANOVA.

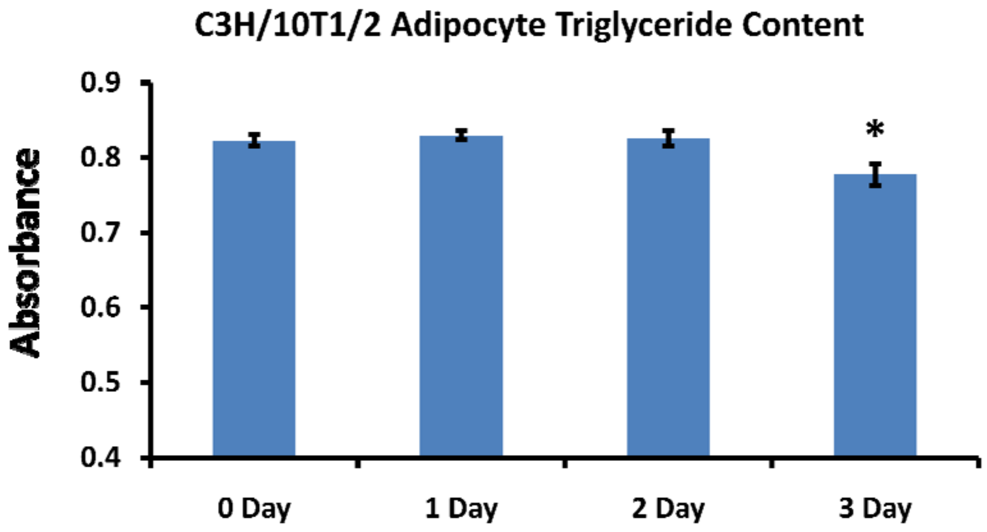

Supplemental Figure 2. Relative mRNA abundance of brown adipose tissue markers Ucp1 and Cidea in C3H/10T1/2 adipocytes and 3T3-L1 adipocytes, determined by QRT-PCR. Data were expressed as –ΔΔCt, equivalent to log2-transformed fold changes (C3H/10T1/2 adipocytes over 3T3-L1 adipocytes). All numerical data were expressed as mean±S.E. *$p < 0.05$ vs 3T3-L1 adipocytes.

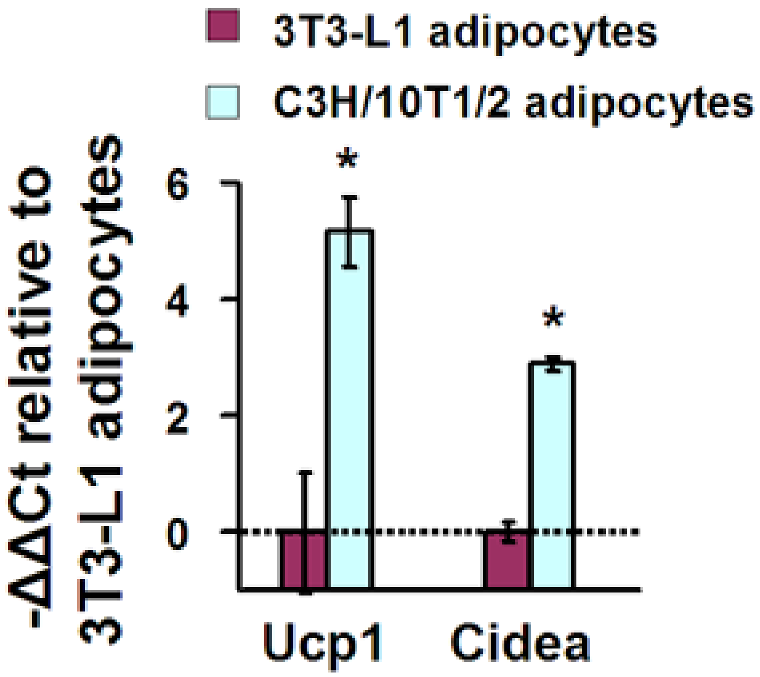

Supplemental Figure 3. Adipsin (Adn), fatty acid synthase (Fasn), and glucose transporter 1 (Slc2a1), were regulated differently by RSG in 3T3-L1 and C3H/10T1/2 adipocytes. Shown are heatmaps of mean fold-changes of probe set intensities in 3T3-L1 and C3H/10T1/2 adipocytes treated with RSG compared to DMSO over time (n = 3 per time point in each cell type). Each row represents one probe set. Glucose transporter 1 (Slc2a1) was represented by 3 probe sets. Only fold-changes significantly different ($p < 0.05$) were shown in colors (red or green).

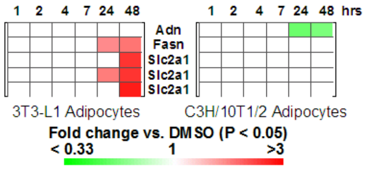

Supplemental Table 1A. Regulation of adipocyte and fibroblast/preadipocyte marker genes with the washout conditions in C3H/10T1/2 cells. Mean intensity values from the microarray experiment were shown (n = 3). One way ANOVA was performed, and $p < 0.05$ was considered significant. For all probesets, the intensities in adipocytes with no washout and adipocytes with 1-day washout were not significantly different from each other, but both were significantly different from the intensities in the respective fibroblast/predipocyte.

Probeset	Gene	Condition	Intensity
1422651_at	Adipoq	Fibroblast/preadipocyte	4
1422651_at	Adipoq	Adipocytes with no washout	5135
1422651_at	Adipoq	Adipocytes with 1-day washout	5271
1417023_a_at	Fabp4	Fibroblast/preadipocyte	25
1417023_a_at	Fabp4	Adipocytes with no washout	5422
1417023_a_at	Fabp4	Adipocytes with 1-day washout	5582
1424155_at	Fabp4	Fibroblast/preadipocyte	3
1424155_at	Fabp4	Adipocytes with no washout	465
1424155_at	Fabp4	Adipocytes with 1-day washout	663
1425809_at	Fabp4	Fibroblast/preadipocyte	16
1425809_at	Fabp4	Adipocytes with no washout	370
1425809_at	Fabp4	Adipocytes with 1-day washout	499
1451263_a_at	Fabp4	Fibroblast/preadipocyte	13
1451263_a_at	Fabp4	Adipocytes with no washout	1627
1451263_a_at	Fabp4	Adipocytes with 1-day washout	1860
1417148_at	Pdgfrb	Fibroblast/preadipocyte	187
1417148_at	Pdgfrb	Adipocytes with no washout	39
1417148_at	Pdgfrb	Adipocytes with 1-day washout	30
1436970_a_at	Pdgfrb	Fibroblast/preadipocyte	386
1436970_a_at	Pdgfrb	Adipocytes with no washout	130
1436970_a_at	Pdgfrb	Adipocytes with 1-day washout	103

Supplemental Table 1B. Regulation of adipocyte and fibroblast/preadipocyte marker genes with the washout conditions in 3T3 L1 cells. Mean intensity values from the microarray experiment were shown (n = 3). One way ANOVA was performed, and $p < 0.05$ was considered significant. For all probesets, the intensities in adipocytes with no

washout and adipocytes with 1-day washout were not significantly different from each other, but both were significantly different from the intensities in the respective fibroblast/predipocyte.

Probeset	Gene	Condition	Intensity
1422651_at	Adipoq	Fibroblast/preadipocyte	8
1422651_at	Adipoq	Adipocytes with no washout	7481
1422651_at	Adipoq	Adipocytes with 1-day washout	7356
1417023_a_at	Fabp4	Fibroblast/preadipocyte	46
1417023_a_at	Fabp4	Adipocytes with no washout	5483
1417023_a_at	Fabp4	Adipocytes with 1-day washout	5123
1424155_at	Fabp4	Fibroblast/preadipocyte	10
1424155_at	Fabp4	Adipocytes with no washout	138
1424155_at	Fabp4	Adipocytes with 1-day washout	139
1425809_at	Fabp4	Fibroblast/preadipocyte	29
1425809_at	Fabp4	Adipocytes with no washout	140
1425809_at	Fabp4	Adipocytes with 1-day washout	162
1451263_a_at	Fabp4	Fibroblast/preadipocyte	16
1451263_a_at	Fabp4	Adipocytes with no washout	1093
1451263_a_at	Fabp4	Adipocytes with 1-day washout	1167
1417148_at	Pdgfrb	Fibroblast/preadipocyte	174
1417148_at	Pdgfrb	Adipocytes with no washout	40
1417148_at	Pdgfrb	Adipocytes with 1-day washout	48
1436970_a_at	Pdgfrb	Fibroblast/preadipocyte	422
1436970_a_at	Pdgfrb	Adipocytes with no washout	114
1436970_a_at	Pdgfrb	Adipocytes with 1-day washout	128

Supplemental Table 1C. Responses of PDK4 and PCK1 to rosiglitazone in C3H/10T1/2 and 3T3-L1 adipocytes. Differentiated C3H/10T1/2 and 3T3-L1 adipocytes were incubated with washout medium for 0, 1, 2, or 3 days and then treated with rosiglitazone (1 μM) or DMSO in the washout medium for 4 hours. Total RNA was extracted from the adipocytes and subject to QRT-PCR after reverse transcription. (1 μM) Fold change. P < 0.05 vs untreated adipocytes with the respective wash-out condition. The relative RNA abundance of PDK4 and PCK1 in rosiglitazone-treated adipocytes was normalized to the housekeeping gene 36B4 (Arbp) and expressed as fold change over DMSO-treated cells with the respective washout days. *P < 0.05 vs DMSO.

		3 day	2 day	1 day	0 day
C3H/10T1/2	PDK4	1.9*	1.8	2.7*	1.3
	PCK1	1.0	1.0	1.4	1.2
3T3-L1	PDK4	1.4	1.1	1.3	1.0
	PCK1	4.2*	5.7*	5.5*	5.7*

Supplemental Table 2. QRT-PCR primer/probe sequences.

Gene name	Forward	Reverse	Probe
Ucp1	TCCTAGGGACCATCACCACC	GCAGGCAGACCGCTGTACA	TGGCAAAAACAGAAGGATTGCCGAAA
Adipoq	CCCAGTCATGCCGAAGATGA	TGCACAAGTTCCCTTGGGTG	TGAAGAGCTAGCTCCTGCTTTGGTCCC
Arbp	GGACCCGAGAAGACCTCCTT	TCAATGGTGCCTCTGGAGATT	CCAGGCTTTGGGCATCACCACG
Opa1	TTGCCTGGGAGACTCTACAAGAG	AATATGTCGTCGTGTTCCTTTCC	TTTCCCGCTTCATGACAGAACCCAA
Ppargc1b	AGGAAGCGGCGGGAAA	CTACAATCTCACCGAACACCTCAA	AGAGATTTCGAATGTATACCACACGGCCTTCA
Scd1	CTGAATGCGAGGGTTGGTTG	CCTGCATGGATCAGCCAAAG	TCCCTGTGCGCTTTGCAAGGTAATGTG
Cs	CCCCTGCCTGAGGGCTTAT	GCCAAGACACCTGTTCCTCTGT	TGGCTGCTGGTAACTGGACAGATGCC
Pdgfrb	GAGGCTTATCCGATGCCTTCT	AGACATGTTGCGAGTAGACAAAACTAA	CAGCGCCGGAGTCACCCAAGGTA
Idh3a	GCACAAAGCTAACATCATGAGGAT	CCGCAACTTCCCTGCATTT	ATGGGCTCTTTCTGC
Cycs	GCAAGCATAAGACTGGACCAAATC	ATGCCTTTGTTCTTGTTGGCATC	TAAGAGAATCCAGCAGCCTGGCCTGTC
Hadha	AAGCTGGACGCCTTGACCAC	GATCTTCTGCTACGTGCTGTGC	TACATCCACACCCACTTCGTCTGCCAG
Pck1	CCACAGCTGCTGCAGAACAC	GAAGGGTCGCATGGCAAA	AGGGCAAGATCATCATGCACGACCC
Pdk4	CAAAGACGGGAAACCCAAGC	CGCAGAGCATCTTTGCACAC	TCGACCCAAACTGTGATGTGGTAGCAG

Supplemental Table 3. Expression of brown adipose tissue markers in 3T3 L1 and C3H/10T1/2 adipocytes. Mean intensity values from the microarray experiment were shown (n = 3) ($p < 0.05$ vs C3H/10T1/2 for the respective probset).

Probeset	Gene	Cell type	Intensity
1418938_at	Dio2	C3H/10T1/2	30
1418938_at	Dio2	3T3 L1	53*
1426081_a_at	Dio2	C3H/10T1/2	19
1426081_a_at	Dio2	3T3 L1	25
1460336_at	Ppargc1a	C3H/10T1/2	56
1460336_at	Ppargc1a	3T3 L1	70

Supplemental Table 4. Regulation of genes by RSG in 3T3-L1 (A) and C3H/10T1/2 (B) adipocytes over time. Each row represents one probe set. The order of the probe sets is identical to those listed in the figures in the manuscript. Certain genes were represented by more than one probe sets. The positive values in the table are fold changes equivalent to mean probe set intensity from RSG-treated divided by that from DMSO-treated adipocytes, and the negative values are fold changes equivalent to mean probe set intensity from DMSO-treated divided by that from RSG-treated adipocytes. Fold-changes not reaching statistical significance ($p < 0.05$) are shown as 0. N = 3 per time point in each cell type.

A. 3T3-L1 Adipocytes

Probe set	Gene Symbol	Category	1 hr	2 hr	4 hr	7 hr	24 hr	48 hr
**Probe sets in Figure 2**								
1415687_a_at	Psap	FAO	0.0	0.0	0.0	0.0	0.0	0.0
1415984_at	Acadm	FAO	0.0	0.0	0.0	0.0	1.3	1.8
1416408_at	Acox1	FAO	0.0	0.0	2.0	1.7	2.2	2.2
1416409_at	Acox1	FAO	0.0	0.0	1.5	1.8	2.6	3.3
1416772_at	Cpt2	FAO	0.0	0.0	0.0	1.3	2.0	2.2
1417008_at	Crat	FAO	0.0	1.3	1.3	1.3	1.8	2.2
1417369_at	Hsd17b4	FAO	0.0	0.0	1.3	1.3	1.6	1.7
1418321_at	Dci	FAO	0.0	0.0	0.0	0.0	1.4	1.3
1418328_at	Cpt1b	FAO	0.0	0.0	0.0	0.0	0.0	1.5
1418862_at	Echdc3	FAO	0.0	0.0	0.0	0.0	0.0	0.0
1419261_at	Acad8	FAO	0.0	0.0	0.0	0.0	0.0	0.0
1419262_at	Acad8	FAO	0.0	0.0	0.0	-1.3	0.0	0.0
1419367_at	Decr1	FAO	0.0	0.0	0.0	0.0	1.6	1.9
1420776_a_at	Auh	FAO	0.0	0.0	0.0	0.0	0.0	0.0
1422526_at	Acsl1	FAO	0.0	2.0	2.4	2.6	2.9	2.9
1423883_at	Acsl1	FAO	0.0	1.5	1.7	1.5	1.6	1.3
1424182_at	Acat1	FAO	0.0	0.0	0.0	0.0	1.2	1.8
1424183_at	Acat1	FAO	0.0	0.0	0.0	0.0	0.0	1.3
1424184_at	Acadvl	FAO	0.0	0.0	0.0	0.0	2.0	2.3
1424451_at	MGC29978	FAO	0.0	0.0	0.0	2.9	11.3	28.4
1425195_a_at	Acat2	FAO	0.0	0.0	0.0	1.5	1.6	2.0
1426522_at	Hadhb	FAO	0.0	0.0	0.0	1.3	3.1	4.0
1428082_at	Acsl5	FAO	0.0	0.0	0.0	0.0	-1.6	0.0
1428145_at	Acaa2	FAO	0.0	0.0	0.0	0.0	1.7	3.0
1428146_s_at	Acaa2	FAO	0.0	0.0	0.0	0.0	1.7	2.6

1429339_a_at	Acad10	FAO	0.0	0.0	0.0	-1.4	0.0	0.0
1429581_at	Acad9	FAO	0.0	0.0	0.0	0.0	0.0	0.0
1435630_s_at	Acat2	FAO	0.0	0.0	1.2	1.4	1.7	2.0
1436756_x_at	Hadhsc	FAO	0.0	0.0	0.0	0.0	0.0	0.0
1437172_x_at	Hadhb	FAO	0.0	0.0	0.0	1.5	1.5	2.5
1438291_x_at	Acads	FAO	0.0	0.0	0.0	0.0	0.0	-1.5
1438391_x_at	Hadh2	FAO	0.0	0.0	0.0	0.0	0.0	-1.2
1448286_at	Hadh2	FAO	0.0	0.0	0.0	0.0	0.0	0.0
1448382_at	Ehhadh	FAO	0.0	0.0	0.0	0.0	0.0	0.0
1448491_at	Ech1	FAO	0.0	0.0	0.0	0.0	1.4	1.7
1448544_at	Crat	FAO	0.0	0.0	1.5	1.4	1.7	1.3
1448717_at	Gcdh	FAO	0.0	0.0	0.0	0.0	0.0	0.0
1448987_at	Acadl	FAO	0.0	0.0	0.0	0.0	1.5	1.7
1448988_at	Acadl	FAO	0.0	0.0	0.0	0.0	0.0	0.0
1449443_at	Decr1	FAO	0.0	0.0	1.2	1.5	2.0	1.6
1449964_a_at	Mlycd	FAO	0.0	0.0	0.0	1.3	2.1	2.6
1450643_s_at	Acsl1	FAO	0.0	1.7	2.0	2.5	2.3	2.1
1451271_a_at	Acat1	FAO	0.0	0.0	0.0	0.0	0.0	1.5
1451511_at	Hibch	FAO	0.0	0.0	0.0	0.0	0.0	0.0
1451512_s_at	Hibch	FAO	0.0	0.0	0.0	0.0	1.2	0.0
1452173_at	Hadha	FAO	0.0	0.0	0.0	0.0	2.6	3.3
1452341_at	Echs1	FAO	0.0	0.0	0.0	0.0	1.3	1.5
1453206_at	Acad9	FAO	0.0	0.0	0.0	0.0	1.5	0.0
1455061_a_at	Acaa2	FAO	0.0	0.0	0.0	0.0	1.5	3.0
1455446_x_at	Acadsb	FAO	0.0	0.0	0.0	-1.2	-1.4	-1.3
1455777_x_at	Hsd17b4	FAO	0.0	0.0	1.3	0.0	1.2	1.4
1455972_x_at	Hadhsc	FAO	0.0	0.0	0.0	0.0	1.4	1.3
1460184_at	Hadhsc	FAO	0.0	0.0	0.0	0.0	1.5	1.7
1460216_at	Acads	FAO	0.0	0.0	0.0	0.0	1.5	1.7
1460316_at	Acsl1	FAO	0.0	1.6	1.4	1.7	1.5	1.7
1460409_at	Cpt1a	FAO	0.0	0.0	0.0	0.0	0.0	0.0
1423972_at	Etfa	OXPHOS/FAO	0.0	0.0	0.0	0.0	1.4	1.5
1428181_at	Etfb	OXPHOS/FAO	0.0	0.0	0.0	0.0	1.6	1.7
1451084_at	Etfdh	OXPHOS/FAO	0.0	0.0	0.0	0.0	2.1	2.2
1429709_at	Pmpcb	OXPHOS	0.0	0.0	0.0	0.0	0.0	0.0
1433729_x_at	Pmpcb	OXPHOS	0.0	0.0	0.0	0.0	0.0	0.0
1434053_x_at	Atp5k	OXPHOS	0.0	0.0	0.0	0.0	1.7	1.5
1450640_x_at	Atp5k	OXPHOS	0.0	0.0	0.0	0.0	0.0	0.0
1415980_at	Atp5g2	OXPHOS	0.0	0.0	0.0	0.0	0.0	0.0
1416058_s_at	Atp5c1	OXPHOS	0.0	0.0	0.0	0.0	1.3	0.0
1416143_at	Atp5j	OXPHOS	0.0	0.0	0.0	1.3	0.0	0.0
1416269_at	Atp5j2	OXPHOS	0.0	0.0	0.0	0.0	1.5	1.5
1416278_a_at	Atp5o	OXPHOS	0.0	0.0	0.0	0.0	1.9	1.6
1416567_s_at	Atp5e	OXPHOS	0.0	0.0	0.0	0.0	0.0	1.3
1416829_at	Atp5b	OXPHOS	0.0	0.0	0.0	0.0	1.3	1.4
1417970_at	Atp5s	OXPHOS	0.0	0.0	0.0	0.0	0.0	-1.4
1420037_at	Atp5a1	OXPHOS	0.0	0.0	0.0	0.0	0.0	0.0
1422508_at	Atp6v1a1	OXPHOS	0.0	0.0	0.0	0.0	0.0	0.0

1423111_at	Atp5a1	OXPHOS	0.0	0.0	0.0	0.0	1.6	1.7
1423676_at	Atp5h	OXPHOS	0.0	0.0	0.0	0.0	1.4	1.2
1423716_s_at	Atp5d	OXPHOS	0.0	0.0	0.0	0.0	1.6	1.4
1426742_at	Atp5f1	OXPHOS	0.0	0.0	0.0	0.0	1.7	1.5
1433562_s_at	Atp5f1	OXPHOS	0.0	0.0	0.0	0.0	1.3	1.5
1435112_a_at	Atp5h	OXPHOS	0.0	0.0	0.0	0.0	0.0	0.0
1435395_s_at	Atp5j2	OXPHOS	0.0	0.0	0.0	0.0	1.3	1.3
1437164_x_at	Atp5o	OXPHOS	0.0	0.0	0.0	0.0	1.3	1.4
1438809_at	Atp5c1	OXPHOS	0.0	0.0	0.0	0.0	0.0	0.0
1448203_at	Atp5l	OXPHOS	0.0	0.0	0.0	0.0	1.4	1.3
1448211_at	0610006O14Rik	OXPHOS	0.0	0.0	0.0	0.0	-1.9	-1.9
1449710_s_at	Atp5a1	OXPHOS	0.0	0.0	0.0	0.0	1.4	1.6
1449712_s_at	Atp6v1e1	OXPHOS	0.0	0.0	0.0	0.0	1.5	1.3
1450634_at	Atp6v1a1	OXPHOS	0.0	0.0	0.0	0.0	0.0	0.0
1454661_at	Atp5g3	OXPHOS	0.0	0.0	0.0	0.0	1.3	0.0
1456580_s_at	Atp5d	OXPHOS	0.0	0.0	0.0	0.0	1.5	1.4
1415966_a_at	Ndufv1	OXPHOS	0.0	0.0	0.0	0.0	1.5	1.6
1415967_at	Ndufv1	OXPHOS	0.0	0.0	0.0	0.0	1.5	1.7
1416056_a_at	Np15	OXPHOS	0.0	0.0	-1.2	0.0	0.0	0.0
1416057_at	Np15	OXPHOS	0.0	0.0	0.0	0.0	1.2	1.2
1416285_at	Ndufc1	OXPHOS	0.0	0.0	0.0	0.0	1.6	1.5
1416366_at	Ndufc2	OXPHOS	0.0	0.0	0.0	1.3	0.0	0.0
1416417_a_at	Ndufb7	OXPHOS	0.0	0.0	0.0	0.0	1.5	1.4
1416495_s_at	Ndufs5	OXPHOS	0.0	0.0	0.0	0.0	1.4	1.2
1416547_at	Ndufb3	OXPHOS	0.0	0.0	0.0	1.4	1.5	1.4
1416663_at	Ndufa9	OXPHOS	0.0	0.0	0.0	0.0	1.2	1.4
1416834_x_at	Ndufb2	OXPHOS	0.0	0.0	0.0	1.3	1.4	1.4
1417102_a_at	Ndufb5	OXPHOS	0.0	0.0	0.0	0.0	1.3	1.4
1417285_a_at	Ndufa5	OXPHOS	0.0	0.0	0.0	0.0	1.5	1.3
1417286_at	Ndufa5	OXPHOS	0.0	0.0	0.0	0.0	1.7	0.0
1417368_s_at	Ndufa2	OXPHOS	0.0	0.0	0.0	0.0	0.0	0.0
1418068_at	Ndufa10	OXPHOS	0.0	0.0	0.0	0.0	0.0	0.0
1418117_at	Ndufs4	OXPHOS	0.0	0.0	0.0	0.0	0.0	0.0
1418814_s_at	2410011G03Rik	OXPHOS	0.0	-1.4	0.0	0.0	1.6	0.0
1422241_a_at	Ndufa1	OXPHOS	0.0	0.0	0.0	0.0	1.3	1.4
1422976_x_at	Ndufa7	OXPHOS	0.0	0.0	0.0	0.0	0.0	0.0
1423692_at	Ndufa8	OXPHOS	0.0	0.0	0.0	1.3	1.6	1.6
1423737_at	Ndufs3	OXPHOS	0.0	0.0	0.0	0.0	1.3	1.2
1423907_a_at	Ndufs8	OXPHOS	0.0	0.0	0.0	0.0	1.4	1.5
1424085_at	Ndufa4	OXPHOS	0.0	0.0	0.0	0.0	1.8	0.0
1424313_a_at	Ndufs7	OXPHOS	0.0	0.0	0.0	0.0	1.2	0.0
1424628_a_at	1500032D16Rik	OXPHOS	0.0	0.0	0.0	0.0	1.4	1.3
1425143_a_at	Ndufs1	OXPHOS	0.0	0.0	0.0	0.0	1.8	1.9
1428075_at	Ndufb4	OXPHOS	0.0	0.0	0.0	0.0	0.0	0.0
1428076_s_at	Ndufb4	OXPHOS	0.0	0.0	0.0	0.0	0.0	0.0
1428159_s_at	Ndufab1	OXPHOS	0.0	0.0	0.0	1.5	1.6	1.3
1428160_at	Ndufab1	OXPHOS	0.0	0.0	0.0	0.0	0.0	1.6
1428179_at	Ndufv2	OXPHOS	0.0	0.0	0.0	0.0	1.5	1.3

1428322_a_at	Ndufb10	OXPHOS	0.0	0.0	0.0	0.0	1.6	1.5
1428360_x_at	Ndufa7	OXPHOS	0.0	0.0	0.0	0.0	0.0	0.0
1428464_at	Ndufa3	OXPHOS	0.0	0.0	0.0	0.0	1.3	1.6
1429708_at	Ndufa11	OXPHOS	0.0	0.0	0.0	0.0	1.6	0.0
1430713_s_at	Grim19	OXPHOS	0.0	0.0	0.0	0.0	0.0	0.0
1433513_x_at	2410011G03Rik	OXPHOS	0.0	0.0	0.0	0.0	1.3	0.0
1434212_at	Ndufs8	OXPHOS	0.0	0.0	0.0	0.0	0.0	1.4
1434213_x_at	Ndufs8	OXPHOS	0.0	0.0	0.0	0.0	0.0	1.3
1434579_x_at	Ndufs8	OXPHOS	0.0	0.0	0.0	0.0	1.4	1.6
1434856_at	Ndufs1	OXPHOS	0.0	0.0	0.0	0.0	0.0	1.3
1435934_at	Ndufab1	OXPHOS	0.0	0.0	0.0	0.0	0.0	0.0
1436567_a_at	Ndufa7	OXPHOS	0.0	0.0	0.0	0.0	0.0	0.0
1436803_a_at	Ndufb9	OXPHOS	0.0	0.0	0.0	0.0	1.4	1.3
1438159_x_at	Ndufv2	OXPHOS	0.0	0.0	0.0	0.0	1.6	1.8
1447919_x_at	Ndufab1	OXPHOS	0.0	0.0	0.0	1.3	1.5	1.5
1448198_a_at	Ndufb8	OXPHOS	0.0	0.0	0.0	0.0	1.4	1.2
1448284_a_at	Ndufc1	OXPHOS	0.0	0.0	0.0	0.0	1.5	1.3
1448331_at	Ndufb7	OXPHOS	0.0	0.0	0.0	1.3	1.5	1.3
1448427_at	Ndufa6	OXPHOS	0.0	0.0	0.0	0.0	1.3	0.0
1448483_a_at	Ndufb2	OXPHOS	0.0	0.0	0.0	0.0	1.3	1.2
1448589_at	Ndufb5	OXPHOS	0.0	0.0	0.0	0.0	1.3	1.4
1448934_at	Ndufa10	OXPHOS	0.0	0.0	0.0	0.0	1.3	0.0
1448959_at	Ndufs4	OXPHOS	0.0	0.0	0.0	0.0	0.0	0.0
1450818_a_at	Ndufa7	OXPHOS	0.0	0.0	0.0	0.0	0.0	0.0
1451096_at	Ndufs2	OXPHOS	0.0	0.0	0.0	0.0	1.2	0.0
1451312_at	Ndufs7	OXPHOS	0.0	0.0	0.0	0.0	1.4	1.3
1452184_at	Ndufb9	OXPHOS	0.0	0.0	0.0	0.0	1.4	1.2
1452692_a_at	Ndufv2	OXPHOS	0.0	0.0	0.0	0.0	1.4	1.3
1452790_x_at	Ndufa3	OXPHOS	0.0	0.0	0.0	0.0	1.5	1.7
1455036_s_at	Ndufc2	OXPHOS	0.0	0.0	0.0	1.2	0.0	0.0
1455283_x_at	Ndufs8	OXPHOS	0.0	0.0	0.0	0.0	1.4	1.5
1455749_x_at	Ndufa7	OXPHOS	0.0	0.0	0.0	0.0	1.3	1.4
1455806_x_at	2410011G03Rik	OXPHOS	0.0	0.0	0.0	0.0	1.4	0.0
1455911_x_at	Np15	OXPHOS	0.0	0.0	0.0	0.0	0.0	1.2
1456015_x_at	Ndufv1	OXPHOS	0.0	0.0	0.0	0.0	1.5	1.7
1415933_a_at	Cox5a	OXPHOS	0.0	0.0	0.0	0.0	1.5	1.5
1415970_at	Cox6c	OXPHOS	0.0	0.0	0.0	0.0	1.3	0.0
1416112_at	Cox8a	OXPHOS	0.0	0.0	0.0	0.0	1.4	1.3
1416565_at	Cox6b1	OXPHOS	0.0	0.0	0.0	0.0	1.7	1.4
1416604_at	Cyc1	OXPHOS	0.0	0.0	0.0	0.0	1.8	1.8
1416902_a_at	Cox5b	OXPHOS	0.0	0.0	0.0	0.0	1.5	1.7
1416970_a_at	Cox7a2	OXPHOS	0.0	0.0	0.0	0.0	1.4	1.4
1416971_at	Cox7a2	OXPHOS	0.0	0.0	0.0	0.0	1.4	1.5
1417417_a_at	Cox6a1	OXPHOS	0.0	0.0	0.0	0.0	1.3	1.3
1417418_s_at	Cox6a1	OXPHOS	0.0	0.0	0.0	0.0	1.3	1.6
1418709_at	Cox7a1	OXPHOS	0.0	0.0	0.0	0.0	2.3	7.2
1426693_x_at	Cox15	OXPHOS	0.0	0.0	0.0	0.0	0.0	1.3
1432264_x_at	Cox7a2l	OXPHOS	0.0	0.0	0.0	0.0	1.3	1.3

1434491_a_at	Cox6c	OXPHOS	0.0	0.0	0.0	0.0	0.0	0.0
1435613_x_at	Cox5b	OXPHOS	0.0	0.0	0.0	0.0	1.5	1.5
1436149_at	Cox5b	OXPHOS	0.0	0.0	-1.4	0.0	1.4	0.0
1436409_at	Cox8a	OXPHOS	0.0	0.0	0.0	0.0	0.0	0.0
1436757_a_at	Cox6b1	OXPHOS	0.0	0.0	0.0	0.0	1.4	1.4
1437982_x_at	Cox15	OXPHOS	0.0	0.0	0.0	0.0	0.0	0.0
1439267_x_at	Cox5a	OXPHOS	0.0	0.0	0.0	1.8	1.7	0.0
1448112_at	Cox7c	OXPHOS	0.0	-1.3	0.0	-1.3	0.0	0.0
1448153_at	Cox5a	OXPHOS	0.0	0.0	0.0	0.0	1.9	1.8
1448222_x_at	Cox8a	OXPHOS	0.0	0.0	0.0	0.0	1.4	1.4
1448322_a_at	Cox4i1	OXPHOS	0.0	0.0	0.0	0.0	0.0	1.2
1449218_at	Cox8b	OXPHOS	0.0	0.0	0.0	0.0	0.0	4.1
1452146_a_at	Cox15	OXPHOS	0.0	0.0	0.0	0.0	0.0	0.0
1454716_x_at	Cox5b	OXPHOS	0.0	0.0	0.0	0.0	1.6	1.7
1455167_at	Cox8c	OXPHOS	0.0	0.0	0.0	0.0	0.0	0.0
1456588_x_at	Cox5b	OXPHOS	0.0	0.0	0.0	0.0	1.4	1.4
1416337_at	Uqcrb	OXPHOS	0.0	0.0	0.0	0.0	1.4	1.6
1424364_a_at	1110020P15Rik	OXPHOS	0.0	0.0	0.0	0.0	2.1	1.8
1427880_at	1500040F11Rik	OXPHOS	0.0	0.0	0.0	0.0	0.0	0.0
1428631_a_at	Uqcrc2	OXPHOS	0.0	0.0	0.0	0.0	1.5	1.3
1428782_a_at	Uqcrc1	OXPHOS	0.0	0.0	0.0	0.0	1.4	1.5
1430326_s_at	1500040F11Rik	OXPHOS	0.0	0.0	0.0	0.0	1.9	2.1
1435757_a_at	Uqcrc2	OXPHOS	0.0	0.0	0.0	0.0	1.4	1.3
1448292_at	Uqcr	OXPHOS	0.0	0.0	0.0	0.0	1.6	1.7
1450968_at	Uqcrfs1	OXPHOS	0.0	0.0	0.0	0.0	1.7	1.5
1452133_at	Uqcrh	OXPHOS	0.0	0.0	0.0	0.0	1.2	1.2
1452613_at	1500040F11Rik	OXPHOS	0.0	0.0	0.0	0.0	0.0	0.0
1455997_a_at	Uqcrb	OXPHOS	0.0	0.0	0.0	0.0	1.5	1.2
1422483_a_at	Cycs	OXPHOS	0.0	0.0	1.6	2.5	3.6	3.2
1422484_at	Cycs	OXPHOS	0.0	0.0	1.6	2.2	3.8	3.3
1418005_at	Sdhb	OXPHOS/TCA	0.0	0.0	0.0	0.0	1.5	1.4
1426688_at	Sdha	OXPHOS/TCA	0.0	0.0	0.0	0.0	1.3	1.5
1426689_s_at	Sdha	OXPHOS/TCA	0.0	0.0	0.0	0.0	1.3	1.5
1428235_at	Sdhd	OXPHOS/TCA	0.0	0.0	0.0	1.3	1.6	1.6
1435986_x_at	Sdhc	OXPHOS/TCA	0.0	0.0	0.0	0.0	1.7	0.0
1437489_x_at	Sdhd	OXPHOS/TCA	0.0	0.0	0.0	0.0	1.5	1.7
1448630_a_at	Sdhc	OXPHOS/TCA	0.0	0.0	0.0	0.0	1.3	0.0
1415891_at	Suclg1	TCA	0.0	0.0	0.0	0.0	1.3	1.3
1416090_at	Pdhb	TCA	0.0	0.0	0.0	1.2	1.8	1.7
1416383_a_at	Pcx	TCA	0.0	0.0	0.0	1.3	2.0	2.0
1416478_a_at	Mdh2	TCA	0.0	0.0	0.0	0.0	1.6	1.6
1416788_a_at	Idh3g	TCA	0.0	0.0	0.0	0.0	1.3	1.3
1416789_at	Idh3g	TCA	0.0	0.0	0.0	0.0	0.0	1.4
1417273_at	Pdk4	TCA	2.5	1.8	0.0	0.0	3.6	6.2
1418560_at	Pdha1	TCA	0.0	0.0	0.0	0.0	1.2	1.5
1418885_a_at	Idh3b	TCA	0.0	0.0	0.0	0.0	1.6	1.6
1418886_s_at	Idh3b	TCA	0.0	0.0	-1.2	0.0	1.5	1.6
1422500_at	Idh3a	TCA	0.0	1.5	1.4	1.6	2.4	2.2

1422501_s_at	Idh3a	TCA	0.0	1.3	1.7	1.9	2.5	2.4
1422577_at	Cs	TCA	0.0	0.0	0.0	0.0	1.9	2.7
1422578_at	Cs	TCA	0.0	0.0	0.0	0.0	1.7	1.8
1423159_at	Dld	TCA	0.0	0.0	0.0	0.0	1.5	1.8
1423710_at	Dlst	TCA	0.0	0.0	0.0	0.0	1.6	2.0
1423747_a_at	Pdk1	TCA	0.0	0.0	0.0	0.0	0.0	2.5
1423748_at	Pdk1	TCA	0.0	0.0	0.0	0.0	1.9	2.6
1424828_a_at	Fh1	TCA	0.0	0.0	0.0	0.0	0.0	1.3
1425615_a_at	1810010O14Rik	TCA	0.0	0.0	0.0	-1.3	-1.5	0.0
1426264_at	Dlat	TCA	0.0	0.0	0.0	0.0	2.2	2.8
1426265_x_at	Dlat	TCA	0.0	0.0	0.0	1.3	2.5	2.9
1426410_at	Pdk3	TCA	0.0	0.0	-1.3	0.0	-1.9	-2.1
1427441_a_at	Suclg2	TCA	0.0	0.0	0.0	0.0	0.0	0.0
1431011_at	Dlst	TCA	0.0	0.0	0.0	0.0	0.0	0.0
1432016_a_at	Idh3a	TCA	0.0	0.0	1.4	1.7	2.2	2.4
1433984_a_at	Mdh2	TCA	0.0	0.0	0.0	0.0	1.2	1.2
1436934_s_at	Aco2	TCA	0.0	0.0	0.0	0.0	2.1	2.5
1448214_at	Pdhb	TCA	0.0	0.0	0.0	1.3	1.9	1.7
1448825_at	Pdk2	TCA	0.0	0.0	0.0	0.0	1.4	1.7
1449137_at	Pdha1	TCA	0.0	0.0	0.0	0.0	1.5	1.6
1450048_a_at	Idh2	TCA	0.0	0.0	0.0	0.0	0.0	0.0
1450667_a_at	Cs	TCA	0.0	0.0	0.0	0.0	1.7	1.9
1451002_at	Aco2	TCA	0.0	0.0	0.0	0.0	1.8	2.0
1451274_at	Ogdh	TCA	0.0	0.0	0.0	0.0	1.9	1.9
1452005_at	Dlat	TCA	0.0	0.0	0.0	0.0	1.4	1.7
1452206_at	Sucla2	TCA	0.0	0.0	0.0	0.0	1.3	1.5
1417956_at	Cidea	UCP	0.0	0.0	0.0	0.0	2.9	7.6
1418197_at	Ucp1	UCP	0.0	0.0	0.0	0.0	0.0	0.0
1420657_at	Ucp3	UCP	0.0	1.6	1.4	1.6	1.4	0.0
1420658_at	Ucp3	UCP	0.0	2.7	0.0	0.0	0.0	1.8
1448188_at	Ucp2	UCP	0.0	0.0	1.3	1.6	1.5	1.5
1416300_a_at	Slc25a3	ATP_Shuttle	0.0	0.0	0.0	0.0	1.3	1.2
1417434_at	Gpd2	ATP_Shuttle	0.0	0.0	0.0	0.0	0.0	0.0
1423108_at	Slc25a20	ATP_Shuttle	0.0	0.0	0.0	0.0	2.1	2.8
1423109_s_at	Slc25a20	ATP_Shuttle	0.0	0.0	0.0	0.0	1.5	1.9
1423772_x_at	Slc25a5	ATP_Shuttle	0.0	0.0	0.0	1.3	1.3	0.0
1424735_at	Slc25a25	ATP_Shuttle	0.0	1.9	2.3	2.3	2.0	2.0
1427483_at	Slc25a24	ATP_Shuttle	0.0	0.0	0.0	-1.8	-2.2	-2.1
1428929_s_at	Slc25a26	ATP_Shuttle	0.0	0.0	0.0	0.0	1.7	2.2
1430542_a_at	Slc25a5	ATP_Shuttle	0.0	0.0	0.0	1.3	1.7	0.0
1434801_x_at	Slc25a5	ATP_Shuttle	0.0	0.0	0.0	0.0	1.4	0.0
1436874_x_at	Slc25a5	ATP_Shuttle	0.0	0.0	0.0	0.0	1.5	0.0
1438360_x_at	Slc25a5	ATP_Shuttle	0.0	0.0	0.0	0.0	0.0	0.0
1438546_x_at	Slc25a5	ATP_Shuttle	0.0	0.0	0.0	1.3	1.4	1.3
1438922_x_at	Slc25a5	ATP_Shuttle	0.0	0.0	0.0	0.0	0.0	0.0
1415990_at	Vdac2	VDAC	0.0	0.0	0.0	0.0	1.4	1.2
1415998_at	Vdac1	VDAC	0.0	0.0	0.0	0.0	1.3	1.6
1416175_a_at	Vdac3	VDAC	0.0	0.0	0.0	0.0	0.0	0.0

1436992_x_at	Vdac1	VDAC	0.0	0.0	0.0	0.0	1.3	1.4
1437192_x_at	Vdac1	VDAC	0.0	0.0	0.0	0.0	1.4	0.0
1437947_x_at	Vdac1	VDAC	0.0	0.0	0.0	0.0	0.0	0.0
**Probe sets in Figure 3A**								
1417066_at	Cabc1	MitHSP	0.0	0.0	0.0	-1.3	0.0	1.6
1417101_at	Hspa2	MitHSP	0.0	0.0	0.0	0.0	0.0	0.0
1417320_at	Grpel1	MitHSP	0.0	0.0	0.0	1.4	1.7	1.5
1418503_at	Hspa9a	MitHSP	0.0	0.0	0.0	0.0	0.0	1.5
1418504_at	Hspa9a	MitHSP	0.0	0.0	0.0	0.0	1.3	1.7
1420629_a_at	Dnaja3	MitHSP	0.0	0.0	0.0	0.0	1.4	1.4
1422579_at	Hspe1	MitHSP	0.0	0.0	0.0	1.3	1.3	0.0
1426351_at	Hspd1	MitHSP	0.0	0.0	0.0	0.0	1.5	1.4
1431274_a_at	Hspa9a	MitHSP	0.0	0.0	0.0	0.0	1.7	0.0
1449935_a_at	Dnaja3	MitHSP	0.0	0.0	0.0	1.2	1.7	1.6
1450668_s_at	Hspe1	MitHSP	0.0	0.0	1.4	1.4	1.4	1.4
1452262_at	Grpel2	MitHSP	0.0	0.0	0.0	0.0	0.0	1.2
1415681_at	Mrpl43	MitRibosomal protein	0.0	0.0	0.0	0.0	1.3	0.0
1415690_at	Mrpl27	MitRibosomal protein	0.0	0.0	0.0	0.0	0.0	0.0
1415762_x_at	Mrpl52	MitRibosomal protein	0.0	0.0	0.0	0.0	-1.3	-1.5
1416284_at	Mrpl28	MitRibosomal protein	0.0	0.0	0.0	0.0	0.0	0.0
1416349_at	Mrpl34	MitRibosomal protein	0.0	0.0	0.0	0.0	1.5	1.4
1416510_at	Mrpl4	MitRibosomal protein	0.0	0.0	0.0	0.0	1.4	0.0
1416595_at	Mrps22	MitRibosomal protein	0.0	0.0	0.0	0.0	0.0	0.0
1416877_a_at	Mrpl51	MitRibosomal protein	0.0	0.0	0.0	0.0	1.4	1.2
1416879_at	Mrpl51	MitRibosomal protein	0.0	0.0	0.0	0.0	0.0	0.0
1416984_at	Mrps18a	MitRibosomal protein	0.0	1.2	0.0	0.0	0.0	-1.3
1417966_at	Mrpl39	MitRibosomal protein	0.0	0.0	0.0	0.0	0.0	0.0
1418112_at	Mrpl10	MitRibosomal protein	0.0	0.0	0.0	0.0	0.0	0.0
1418137_at	Mrp63	MitRibosomal protein	0.0	0.0	0.0	0.0	0.0	0.0
1418716_at	Mrps25	MitRibosomal protein	0.0	-1.5	0.0	0.0	-1.3	-1.3
1419362_at	Mrpl35	MitRibosomal protein	1.7	0.0	0.0	0.0	0.0	0.0
1419363_a_at	Mrpl35	MitRibosomal protein	0.0	0.0	0.0	0.0	0.0	0.0
1420488_at	Mrps14	MitRibosomal protein	0.0	0.0	0.0	0.0	0.0	1.5
1420489_at	Mrps14	MitRibosomal protein	0.0	0.0	0.0	0.0	0.0	0.0
1420845_at	Mrps2	MitRibosomal protein	0.0	0.0	0.0	0.0	0.0	1.6
1420846_at	Mrps2	MitRibosomal protein	0.0	0.0	1.3	1.3	1.3	1.5
1421874_a_at	Mrps23	MitRibosomal protein	0.0	0.0	0.0	0.0	0.0	1.5
1421875_a_at	Mrps23	MitRibosomal protein	0.0	0.0	0.0	0.0	1.3	1.4
1421914_s_at	Mrpl19	MitRibosomal protein	0.0	0.0	0.0	0.0	0.0	0.0
1421971_a_at	Mrps34	MitRibosomal protein	0.0	0.0	0.0	1.3	1.5	1.3
1422451_at	Mrps21	MitRibosomal protein	0.0	0.0	0.0	0.0	0.0	0.0
1422819_at	Mrpl36	MitRibosomal protein	0.0	0.0	0.0	0.0	0.0	0.0
1423219_a_at	Mrpl49	MitRibosomal protein	0.0	0.0	0.0	0.0	1.2	0.0
1423242_at	Mrps36	MitRibosomal protein	0.0	0.0	0.0	0.0	0.0	0.0
1423492_at	Mrpl45	MitRibosomal protein	0.0	0.0	0.0	0.0	1.4	0.0
1423764_s_at	Mrpl37	MitRibosomal protein	0.0	0.0	0.0	0.0	0.0	0.0
1423857_at	Mrpl30	MitRibosomal protein	0.0	0.0	0.0	0.0	0.0	0.0
1424164_at	Mrpl50	MitRibosomal protein	0.0	0.0	0.0	0.0	0.0	0.0

1424204_at	Mrpl13	MitRibosomal protein	0.0	0.0	0.0	0.0	0.0	-1.3
1424372_at	Mrpl32	MitRibosomal protein	0.0	0.0	0.0	0.0	0.0	0.0
1424440_at	Mrps6	MitRibosomal protein	0.0	0.0	0.0	0.0	0.0	-1.6
1425189_a_at	Mrpl15	MitRibosomal protein	0.0	0.0	0.0	0.0	1.5	0.0
1426651_at	Mrpl44	MitRibosomal protein	0.0	0.0	0.0	0.0	0.0	0.0
1427158_at	Mrps30	MitRibosomal protein	0.0	0.0	0.0	0.0	0.0	0.0
1427173_a_at	Mrps33	MitRibosomal protein	0.0	0.0	0.0	0.0	0.0	0.0
1427297_at	Mrpl9	MitRibosomal protein	0.0	0.0	0.0	0.0	0.0	0.0
1427901_at	Mrps18c	MitRibosomal protein	0.0	0.0	0.0	0.0	0.0	0.0
1428302_at	Mrpl48	MitRibosomal protein	0.0	0.0	0.0	0.0	0.0	0.0
1428709_a_at	Mrpl24	MitRibosomal protein	0.0	0.0	0.0	0.0	0.0	-1.3
1429054_at	Mrpl47	MitRibosomal protein	0.0	0.0	0.0	1.5	1.6	0.0
1429453_a_at	Mrpl55	MitRibosomal protein	0.0	0.0	0.0	1.3	1.4	1.8
1430976_a_at	Mrpl9	MitRibosomal protein	0.0	0.0	0.0	0.0	0.0	1.3
1433878_at	Mrps26	MitRibosomal protein	0.0	0.0	0.0	1.3	0.0	0.0
1434971_x_at	Mrpl15	MitRibosomal protein	0.0	0.0	0.0	0.0	1.7	1.6
1435232_x_at	Mrpl15	MitRibosomal protein	0.0	0.0	0.0	0.0	1.3	1.4
1435843_x_at	Mrps9	MitRibosomal protein	0.0	0.0	0.0	0.0	0.0	0.0
1435966_x_at	Mrpl13	MitRibosomal protein	0.0	0.0	0.0	0.0	0.0	0.0
1435995_at	Mrpl22	MitRibosomal protein	0.0	0.0	0.0	0.0	0.0	-1.3
1437131_x_at	Mrpl11	MitRibosomal protein	0.0	0.0	0.0	0.0	0.0	0.0
1437391_x_at	Mrpl44	MitRibosomal protein	0.0	-1.2	0.0	0.0	0.0	0.0
1437622_x_at	Mrpl28	MitRibosomal protein	0.0	0.0	0.0	0.0	0.0	0.0
1437839_x_at	Mrpl11	MitRibosomal protein	0.0	0.0	0.0	0.0	0.0	0.0
1437997_x_at	Mrpl48	MitRibosomal protein	0.0	0.0	0.0	0.0	0.0	0.0
1438014_at	Mrpl34	MitRibosomal protein	0.0	0.0	0.0	0.0	0.0	0.0
1438563_s_at	Mrps24	MitRibosomal protein	0.0	0.0	0.0	0.0	0.0	0.0
1439210_at	Mrps9	MitRibosomal protein	0.0	4.2	0.0	0.0	0.0	0.0
1447961_s_at	Mrpl38	MitRibosomal protein	0.0	0.0	0.0	0.0	0.0	0.0
1448373_at	Mrpl18	MitRibosomal protein	0.0	0.0	0.0	1.3	1.5	1.2
1448488_at	Mrps5	MitRibosomal protein	0.0	0.0	0.0	1.3	0.0	0.0
1448699_at	Mrpl54	MitRibosomal protein	0.0	0.0	0.0	0.0	0.0	0.0
1448799_s_at	Mrps12	MitRibosomal protein	0.0	0.0	0.0	0.0	0.0	0.0
1448811_at	Mrpl2	MitRibosomal protein	-1.3	0.0	0.0	0.0	0.0	0.0
1448849_at	Mrpl40	MitRibosomal protein	0.0	0.0	0.0	-1.3	0.0	0.0
1448869_a_at	Mrps16	MitRibosomal protein	0.0	0.0	0.0	0.0	1.3	0.0
1448909_a_at	Mrpl39	MitRibosomal protein	0.0	0.0	0.0	0.0	0.0	0.0
1448921_a_at	Mrps9	MitRibosomal protein	0.0	0.0	0.0	-1.2	0.0	0.0
1449004_at	Mrpl46	MitRibosomal protein	0.0	0.0	0.0	0.0	1.3	0.0
1449194_at	Mrps25	MitRibosomal protein	0.0	0.0	0.0	0.0	0.0	0.0
1449294_at	Mrps15	MitRibosomal protein	0.0	0.0	0.0	0.0	0.0	-2.1
1450405_at	Mrpl19	MitRibosomal protein	0.0	0.0	0.0	0.0	0.0	0.0
1450866_a_at	Mrpl17	MitRibosomal protein	0.0	0.0	0.0	0.0	-1.2	0.0
1450867_at	Mrpl17	MitRibosomal protein	0.0	0.0	0.0	0.0	0.0	0.0
1450880_at	Mrpl16	MitRibosomal protein	0.0	0.0	0.0	0.0	0.0	0.0
1450948_a_at	Mrpl1	MitRibosomal protein	0.0	0.0	0.0	0.0	0.0	0.0
1451164_a_at	Mrps18b	MitRibosomal protein	-1.2	0.0	0.0	1.3	1.4	1.5
1451178_at	Mrpl53	MitRibosomal protein	0.0	0.0	0.0	0.0	1.3	0.0

1451266_at	Mrpl50	MitRibosomal protein	0.0	0.0	0.0	0.0	0.0	0.0
1451307_at	Mrpl14	MitRibosomal protein	0.0	0.0	0.0	0.0	0.0	0.0
1452048_at	Mrpl12	MitRibosomal protein	0.0	0.0	0.0	1.4	2.0	2.0
1452144_a_at	Mrpl44	MitRibosomal protein	0.0	0.0	0.0	0.0	0.0	0.0
1452585_at	Mrps28	MitRibosomal protein	-1.3	0.0	0.0	0.0	1.3	1.2
1453571_at	Mrpl19	MitRibosomal protein	0.0	0.0	0.0	0.0	0.0	0.0
1453725_a_at	Mrps7	MitRibosomal protein	0.0	0.0	0.0	0.0	0.0	0.0
1453728_a_at	Mrps17	MitRibosomal protein	0.0	0.0	0.0	0.0	0.0	0.0
1453954_a_at	Mrps5	MitRibosomal protein	0.0	0.0	0.0	0.0	0.0	0.0
1456109_a_at	Mrps15	MitRibosomal protein	0.0	0.0	0.0	0.0	0.0	-1.6
1456313_x_at	Mrpl28	MitRibosomal protein	0.0	0.0	0.0	0.0	0.0	0.0
1460195_at	Mrps11	MitRibosomal protein	0.0	0.0	0.0	0.0	0.0	0.0
1460225_at	Mrp63	MitRibosomal protein	0.0	0.0	0.0	0.0	0.0	0.0
1460354_a_at	Mrpl13	MitRibosomal protein	0.0	0.0	0.0	0.0	0.0	0.0
1460701_a_at	Mrpl52	MitRibosomal protein	0.0	0.0	0.0	0.0	-1.4	-2.1
1416345_at	Timm8a	TIMMS & TOMMS	0.0	0.0	0.0	1.9	1.9	2.1
1416346_at	Timm8a	TIMMS & TOMMS	0.0	0.0	0.0	1.8	1.8	1.8
1416485_at	Timm23	TIMMS & TOMMS	0.0	0.0	0.0	1.5	1.7	1.5
1417192_at	Tomm70a	TIMMS & TOMMS	0.0	0.0	0.0	1.6	1.5	1.7
1417499_at	Timm13a	TIMMS & TOMMS	0.0	0.0	1.3	1.4	1.4	1.3
1417670_at	Timm44	TIMMS & TOMMS	0.0	0.0	0.0	0.0	1.4	1.7
1417916_a_at	Fxc1	TIMMS & TOMMS	0.0	0.0	0.0	1.4	1.4	0.0
1423079_a_at	Tomm20	TIMMS & TOMMS	0.0	0.0	0.0	0.0	0.0	0.0
1423080_at	Tomm20	TIMMS & TOMMS	0.0	0.0	0.0	0.0	0.0	0.0
1423081_a_at	Tomm20	TIMMS & TOMMS	0.0	0.0	0.0	1.2	0.0	0.0
1426118_a_at	Tomm40	TIMMS & TOMMS	0.0	0.0	0.0	0.0	1.4	1.5
1426256_at	Timm17a	TIMMS & TOMMS	0.0	0.0	0.0	0.0	1.3	0.0
1426675_at	Tomm70a	TIMMS & TOMMS	0.0	0.0	0.0	1.5	1.6	1.6
1426676_s_at	Tomm70a	TIMMS & TOMMS	0.0	0.0	1.4	1.7	1.5	1.8
1428214_at	Tomm7	TIMMS & TOMMS	0.0	0.0	0.0	0.0	0.0	0.0
1428215_x_at	Tomm7	TIMMS & TOMMS	0.0	0.0	0.0	0.0	0.0	0.0
1428216_s_at	Tomm7	TIMMS & TOMMS	0.0	0.0	0.0	0.0	0.0	0.0
1431665_a_at	Timm8b	TIMMS & TOMMS	0.0	0.0	0.0	0.0	0.0	0.0
1434732_x_at	Tomm7	TIMMS & TOMMS	0.0	0.0	0.0	0.0	0.0	0.0
1435534_a_at	Tomm20	TIMMS & TOMMS	0.0	0.0	0.0	0.0	0.0	0.0
1438656_x_at	Timm17b	TIMMS & TOMMS	0.0	0.0	0.0	0.0	0.0	0.0
1439371_x_at	Timm44	TIMMS & TOMMS	0.0	0.0	0.0	0.0	0.0	0.0
1448345_at	Tomm34	TIMMS & TOMMS	0.0	0.0	0.0	0.0	0.0	0.0
1448517_at	Timm22	TIMMS & TOMMS	0.0	0.0	0.0	0.0	1.5	0.0
1448801_a_at	Timm44	TIMMS & TOMMS	0.0	0.0	0.0	0.0	0.0	1.3
1449886_a_at	Timm10	TIMMS & TOMMS	0.0	0.0	0.0	1.5	1.7	0.0
1455211_a_at	Timm9	TIMMS & TOMMS	0.0	0.0	0.0	0.0	1.5	1.5
1455357_x_at	Tomm20	TIMMS & TOMMS	0.0	0.0	0.0	1.3	0.0	0.0
1455456_a_at	Timm50	TIMMS & TOMMS	0.0	0.0	0.0	0.0	0.0	0.0
1460685_at	Timm17b	TIMMS & TOMMS	0.0	0.0	0.0	0.0	0.0	0.0
**Probe sets in Figure 3B**								
1429533_at	Immt	Structure	0.0	0.0	0.0	0.0	1.7	2.3
1429534_a_at	Immt	Structure	0.0	0.0	0.0	0.0	1.5	1.6

1433470_a_at	Immt	Structure	0.0	0.0	0.0	0.0	1.5	1.5
1418768_at	Opa1	Structure	0.0	0.0	0.0	0.0	0.0	1.7
1449214_a_at	Opa1	Structure	0.0	0.0	0.0	0.0	1.9	2.2
1451541_at	Bcs1l	Structure	0.0	0.0	0.0	0.0	0.0	0.0
1450561_a_at	Surf1	Structure	0.0	0.0	0.0	0.0	-1.5	-1.7
**Probe sets in Figure 3C**								
1460652_at	Esrra	Transcription Factor	0.0	0.0	0.0	0.0	1.7	1.6
1450664_at	Gabpa	Transcription Factor	0.0	0.0	0.0	0.0	0.0	0.0
1450665_at	Gabpa	Transcription Factor	0.0	0.0	0.0	0.0	1.4	1.5
1436232_a_at	Gabpb1	Transcription Factor	0.0	-1.4	0.0	0.0	0.0	0.0
1419761_a_at	Gabpb1	Transcription Factor	0.0	0.0	0.0	0.0	0.0	0.0
1450444_a_at	Nr1h3	Transcription factor	0.0	0.0	0.0	0.0	0.0	0.0
1424787_a_at	Nrf1	Transcription Factor	0.0	0.0	1.3	0.0	0.0	0.0
1434627_at	Nrf1	Transcription Factor	0.0	0.0	0.0	0.0	0.0	0.0
1418469_at	Nrip1	Transcription Factor	0.0	1.5	1.4	1.4	1.5	0.0
1449089_at	Nrip1	Transcription Factor	0.0	1.4	1.4	1.5	1.3	0.0
1449051_at	Ppara	Transcription Factor	0.0	0.0	0.0	0.0	5.9	6.4
1460336_at	Ppargc1a	Transcription Factor	0.0	0.0	0.0	0.0	2.2	0.0
1449945_at	Ppargc1b	Transcription Factor	0.0	1.9	1.8	1.7	2.1	2.0
1426381_at	Pprc1	Transcription Factor	0.0	0.0	0.0	0.0	0.0	0.0
1448224_at	Tfam	Transcription Factor	0.0	0.0	0.0	0.0	-1.2	0.0
1449749_s_at	Tfb1m	Transcription Factor	0.0	0.0	0.0	-1.3	-1.4	0.0
1423441_at	Tfb2m	Transcription Factor	0.0	0.0	0.0	0.0	0.0	0.0
**Probe sets in Supplemental Figure 2**								
1417867_at	Adn		0.0	0.0	0.0	0.0	0.0	0.0
1423828_at	Fasn		0.0	0.0	0.0	0.0	1.4	1.7
1434773_a_at	Slc2a1		0.0	0.0	0.0	0.0	0.0	2.7
1426600_at	Slc2a1		0.0	0.0	0.0	0.0	1.5	2.4
1426599_a_at	Slc2a1		0.0	0.0	0.0	0.0	0.0	2.5
**Probe sets mentioned in Discussion**								
1422820_at	Lipe		0.0	0.0	1.4	0.0	1.3	1.3
1428143_a_at	Pnpla2		0.0	0.0	0.0	1.4	1.7	1.6
1428591_at	Pnpla2		0.0	0.0	0.0	1.6	1.8	1.7
1427367_at	Pnpla2		0.0	0.0	0.0	0.0	0.0	0.0

B. C3H/10T1/2 Adipocytes

Probe set	Gene Symbol	Category	1 hr	2 hr	4 hr	7 hr	24 hr	48 hr
**Probe sets in Figure 2**								
1415687_a_at	Psap	FAO	0.0	0.0	0.0	0.0	0.0	0.0
1415984_at	Acadm	FAO	0.0	0.0	0.0	1.2	1.6	1.8
1416408_at	Acox1	FAO	0.0	0.0	0.0	1.4	0.0	1.4
1416409_at	Acox1	FAO	0.0	0.0	0.0	1.5	1.6	1.6
1416772_at	Cpt2	FAO	0.0	0.0	0.0	0.0	1.3	1.4
1417008_at	Crat	FAO	0.0	1.2	1.2	1.4	1.5	1.7
1417369_at	Hsd17b4	FAO	0.0	0.0	1.3	1.3	1.5	1.3
1418321_at	Dci	FAO	0.0	0.0	0.0	0.0	1.5	1.5

1418328_at	Cpt1b	FAO	0.0	0.0	0.0	0.0	4.8	6.0
1418862_at	Echdc3	FAO	0.0	0.0	0.0	0.0	0.0	0.0
1419261_at	Acad8	FAO	0.0	0.0	0.0	0.0	0.0	0.0
1419262_at	Acad8	FAO	0.0	0.0	0.0	0.0	0.0	1.2
1419367_at	Decr1	FAO	0.0	0.0	0.0	0.0	1.5	1.7
1420776_a_at	Auh	FAO	0.0	0.0	0.0	0.0	0.0	0.0
1422526_at	Acsl1	FAO	0.0	1.3	1.7	3.1	1.3	1.3
1423883_at	Acsl1	FAO	0.0	0.0	0.0	1.9	1.2	0.0
1424182_at	Acat1	FAO	0.0	0.0	0.0	0.0	1.8	1.3
1424183_at	Acat1	FAO	0.0	0.0	0.0	0.0	0.0	1.3
1424184_at	Acadvl	FAO	0.0	0.0	0.0	0.0	1.6	1.8
1424451_at	MGC29978	FAO	0.0	0.0	1.2	1.4	2.8	3.6
1425195_a_at	Acat2	FAO	0.0	0.0	0.0	0.0	1.4	0.0
1426522_at	Hadhb	FAO	0.0	0.0	0.0	0.0	1.9	2.1
1428082_at	Acsl5	FAO	0.0	0.0	0.0	0.0	-1.6	-1.3
1428145_at	Acaa2	FAO	0.0	0.0	0.0	0.0	1.9	3.1
1428146_s_at	Acaa2	FAO	0.0	0.0	0.0	0.0	1.9	2.5
1429339_a_at	Acad10	FAO	0.0	0.0	0.0	0.0	0.0	1.5
1429581_at	Acad9	FAO	0.0	0.0	0.0	0.0	0.0	0.0
1435630_s_at	Acat2	FAO	0.0	0.0	0.0	0.0	1.3	0.0
1436756_x_at	Hadhsc	FAO	0.0	0.0	0.0	0.0	0.0	0.0
1437172_x_at	Hadhb	FAO	0.0	0.0	0.0	0.0	1.8	1.6
1438291_x_at	Acads	FAO	0.0	0.0	0.0	0.0	0.0	0.0
1438391_x_at	Hadh2	FAO	0.0	0.0	0.0	0.0	0.0	0.0
1448286_at	Hadh2	FAO	0.0	0.0	0.0	0.0	0.0	0.0
1448382_at	Ehhadh	FAO	0.0	2.6	2.3	2.8	2.7	2.4
1448491_at	Ech1	FAO	0.0	0.0	0.0	0.0	1.2	1.4
1448544_at	Crat	FAO	0.0	0.0	0.0	0.0	0.0	0.0
1448717_at	Gcdh	FAO	0.0	0.0	0.0	0.0	0.0	0.0
1448987_at	Acadl	FAO	0.0	0.0	0.0	1.3	1.5	1.3
1448988_at	Acadl	FAO	0.0	0.0	0.0	0.0	0.0	0.0
1449443_at	Decr1	FAO	0.0	0.0	0.0	1.3	1.5	1.4
1449964_a_at	Mlycd	FAO	0.0	0.0	0.0	1.5	1.7	1.4
1450643_s_at	Acsl1	FAO	0.0	0.0	1.6	2.7	0.0	0.0
1451271_a_at	Acat1	FAO	0.0	0.0	0.0	0.0	0.0	1.4
1451511_at	Hibch	FAO	0.0	0.0	0.0	0.0	0.0	0.0
1451512_s_at	Hibch	FAO	0.0	0.0	0.0	0.0	0.0	0.0
1452173_at	Hadha	FAO	0.0	0.0	0.0	1.2	1.9	2.0
1452341_at	Echs1	FAO	0.0	0.0	0.0	0.0	0.0	0.0
1453206_at	Acad9	FAO	0.0	0.0	0.0	0.0	0.0	0.0
1455061_a_at	Acaa2	FAO	0.0	0.0	0.0	1.4	2.0	2.3
1455446_x_at	Acadsb	FAO	0.0	0.0	0.0	-1.3	-1.5	-1.4
1455777_x_at	Hsd17b4	FAO	0.0	0.0	0.0	0.0	1.4	0.0
1455972_x_at	Hadhsc	FAO	0.0	0.0	0.0	0.0	1.3	0.0
1460184_at	Hadhsc	FAO	0.0	0.0	0.0	0.0	1.3	1.3
1460216_at	Acads	FAO	0.0	0.0	1.2	0.0	1.3	1.5
1460316_at	Acsl1	FAO	0.0	0.0	1.7	1.9	1.3	0.0
1460409_at	Cpt1a	FAO	0.0	0.0	0.0	0.0	0.0	0.0

1423972_at	Etfa	OXPHOS/FAO	0.0	0.0	0.0	0.0	0.0	1.3
1428181_at	Etfb	OXPHOS/FAO	0.0	0.0	0.0	0.0	0.0	1.3
1451084_at	Etfdh	OXPHOS/FAO	0.0	0.0	0.0	1.3	1.8	1.9
1429709_at	Pmpcb	OXPHOS	0.0	0.0	0.0	0.0	0.0	0.0
1433729_x_at	Pmpcb	OXPHOS	0.0	0.0	0.0	0.0	0.0	0.0
1434053_x_at	Atp5k	OXPHOS	0.0	0.0	0.0	0.0	1.6	1.4
1450640_x_at	Atp5k	OXPHOS	0.0	0.0	0.0	0.0	1.5	1.4
1415980_at	Atp5g2	OXPHOS	0.0	0.0	0.0	0.0	0.0	0.0
1416058_s_at	Atp5c1	OXPHOS	0.0	0.0	0.0	0.0	0.0	1.2
1416143_at	Atp5j	OXPHOS	0.0	0.0	0.0	0.0	1.3	0.0
1416269_at	Atp5j2	OXPHOS	0.0	0.0	0.0	1.2	1.6	0.0
1416278_a_at	Atp5o	OXPHOS	0.0	0.0	0.0	0.0	1.7	1.3
1416567_s_at	Atp5e	OXPHOS	0.0	0.0	0.0	0.0	1.4	0.0
1416829_at	Atp5b	OXPHOS	0.0	0.0	0.0	0.0	0.0	0.0
1417970_at	Atp5s	OXPHOS	0.0	0.0	0.0	0.0	0.0	0.0
1420037_at	Atp5a1	OXPHOS	0.0	0.0	0.0	0.0	0.0	0.0
1422508_at	Atp6v1a1	OXPHOS	0.0	0.0	0.0	0.0	0.0	0.0
1423111_at	Atp5a1	OXPHOS	0.0	0.0	0.0	0.0	1.3	1.4
1423676_at	Atp5h	OXPHOS	0.0	0.0	0.0	0.0	1.2	1.2
1423716_s_at	Atp5d	OXPHOS	0.0	0.0	0.0	0.0	1.4	1.3
1426742_at	Atp5f1	OXPHOS	0.0	0.0	0.0	0.0	1.3	1.4
1433562_s_at	Atp5f1	OXPHOS	0.0	0.0	0.0	0.0	1.4	0.0
1435112_a_at	Atp5h	OXPHOS	0.0	0.0	0.0	0.0	0.0	1.5
1435395_s_at	Atp5j2	OXPHOS	0.0	0.0	0.0	0.0	1.3	0.0
1437164_x_at	Atp5o	OXPHOS	0.0	0.0	0.0	0.0	1.4	1.3
1438809_at	Atp5c1	OXPHOS	0.0	0.0	0.0	0.0	0.0	0.0
1448203_at	Atp5l	OXPHOS	0.0	0.0	0.0	0.0	1.4	0.0
1448211_at	0610006O14Rik	OXPHOS	0.0	0.0	0.0	0.0	-1.5	0.0
1449710_s_at	Atp5a1	OXPHOS	0.0	0.0	0.0	0.0	1.3	1.3
1449712_s_at	Atp6v1e1	OXPHOS	0.0	0.0	0.0	1.2	0.0	0.0
1450634_at	Atp6v1a1	OXPHOS	0.0	0.0	0.0	0.0	0.0	0.0
1454661_at	Atp5g3	OXPHOS	0.0	0.0	0.0	0.0	0.0	0.0
1456580_s_at	Atp5d	OXPHOS	0.0	0.0	0.0	0.0	1.3	1.3
1415966_a_at	Ndufv1	OXPHOS	0.0	0.0	0.0	0.0	1.4	1.4
1415967_at	Ndufv1	OXPHOS	0.0	0.0	0.0	0.0	1.4	1.4
1416056_a_at	Np15	OXPHOS	0.0	0.0	0.0	0.0	0.0	1.2
1416057_at	Np15	OXPHOS	0.0	0.0	0.0	0.0	1.3	1.2
1416285_at	Ndufc1	OXPHOS	0.0	0.0	0.0	0.0	1.4	1.4
1416366_at	Ndufc2	OXPHOS	0.0	0.0	0.0	1.3	1.5	1.4
1416417_a_at	Ndufb7	OXPHOS	0.0	0.0	0.0	0.0	1.4	1.3
1416495_s_at	Ndufs5	OXPHOS	0.0	0.0	0.0	0.0	1.4	0.0
1416547_at	Ndufb3	OXPHOS	0.0	0.0	1.2	1.4	1.6	1.4
1416663_at	Ndufa9	OXPHOS	0.0	0.0	0.0	0.0	1.4	1.4
1416834_x_at	Ndufb2	OXPHOS	0.0	0.0	0.0	0.0	0.0	0.0
1417102_a_at	Ndufb5	OXPHOS	0.0	0.0	0.0	0.0	1.6	0.0
1417285_a_at	Ndufa5	OXPHOS	0.0	0.0	0.0	0.0	1.6	1.4
1417286_at	Ndufa5	OXPHOS	0.0	0.0	0.0	1.3	1.5	1.4
1417368_s_at	Ndufa2	OXPHOS	0.0	0.0	0.0	0.0	1.3	0.0

1418068_at	Ndufa10	OXPHOS	0.0	0.0	0.0	0.0	2.1	0.0
1418117_at	Ndufs4	OXPHOS	0.0	0.0	0.0	0.0	0.0	0.0
1418814_s_at	2410011G03Rik	OXPHOS	0.0	0.0	0.0	0.0	1.5	0.0
1422241_a_at	Ndufa1	OXPHOS	0.0	0.0	0.0	0.0	1.2	0.0
1422976_x_at	Ndufa7	OXPHOS	0.0	0.0	0.0	0.0	1.5	0.0
1423692_at	Ndufa8	OXPHOS	0.0	0.0	0.0	1.3	1.4	1.5
1423737_at	Ndufs3	OXPHOS	0.0	0.0	0.0	0.0	1.3	1.3
1423907_a_at	Ndufs8	OXPHOS	0.0	0.0	0.0	0.0	1.3	1.3
1424085_at	Ndufa4	OXPHOS	0.0	0.0	0.0	0.0	1.4	1.3
1424313_a_at	Ndufs7	OXPHOS	0.0	0.0	0.0	0.0	1.4	1.5
1424628_a_at	1500032D16Rik	OXPHOS	0.0	0.0	0.0	0.0	0.0	1.3
1425143_a_at	Ndufs1	OXPHOS	0.0	0.0	0.0	0.0	1.4	1.7
1428075_at	Ndufb4	OXPHOS	0.0	0.0	0.0	0.0	1.3	0.0
1428076_s_at	Ndufb4	OXPHOS	0.0	0.0	0.0	0.0	1.3	0.0
1428159_s_at	Ndufab1	OXPHOS	0.0	0.0	0.0	0.0	1.4	1.3
1428160_at	Ndufab1	OXPHOS	0.0	0.0	0.0	0.0	0.0	0.0
1428179_at	Ndufv2	OXPHOS	0.0	0.0	0.0	1.2	1.3	0.0
1428322_a_at	Ndufb10	OXPHOS	0.0	0.0	0.0	1.4	1.6	1.6
1428360_x_at	Ndufa7	OXPHOS	0.0	0.0	0.0	0.0	1.2	1.3
1428464_at	Ndufa3	OXPHOS	0.0	0.0	0.0	0.0	1.3	1.3
1429708_at	Ndufa11	OXPHOS	0.0	0.0	0.0	0.0	1.4	0.0
1430713_s_at	Grim19	OXPHOS	0.0	0.0	0.0	0.0	1.3	1.3
1433513_x_at	2410011G03Rik	OXPHOS	0.0	0.0	0.0	0.0	0.0	0.0
1434212_at	Ndufs8	OXPHOS	0.0	0.0	0.0	0.0	0.0	0.0
1434213_x_at	Ndufs8	OXPHOS	0.0	0.0	0.0	0.0	1.5	1.3
1434579_x_at	Ndufs8	OXPHOS	0.0	0.0	1.3	0.0	1.3	1.5
1434856_at	Ndufs1	OXPHOS	0.0	0.0	0.0	1.7	1.9	1.6
1435934_at	Ndufab1	OXPHOS	0.0	0.0	0.0	0.0	0.0	0.0
1436567_a_at	Ndufa7	OXPHOS	0.0	0.0	0.0	0.0	0.0	0.0
1436803_a_at	Ndufb9	OXPHOS	0.0	0.0	0.0	0.0	1.3	0.0
1438159_x_at	Ndufv2	OXPHOS	0.0	0.0	0.0	1.4	1.6	1.4
1447919_x_at	Ndufab1	OXPHOS	0.0	0.0	0.0	0.0	1.4	0.0
1448198_a_at	Ndufb8	OXPHOS	0.0	0.0	0.0	0.0	1.5	1.3
1448284_a_at	Ndufc1	OXPHOS	0.0	0.0	0.0	0.0	1.6	1.4
1448331_at	Ndufb7	OXPHOS	0.0	0.0	0.0	0.0	0.0	1.4
1448427_at	Ndufa6	OXPHOS	0.0	0.0	0.0	0.0	1.3	1.3
1448483_a_at	Ndufb2	OXPHOS	0.0	0.0	0.0	0.0	0.0	0.0
1448589_at	Ndufb5	OXPHOS	0.0	0.0	0.0	0.0	1.6	1.4
1448934_at	Ndufa10	OXPHOS	0.0	0.0	0.0	0.0	1.5	1.4
1448959_at	Ndufs4	OXPHOS	0.0	0.0	0.0	0.0	0.0	1.2
1450818_a_at	Ndufa7	OXPHOS	0.0	0.0	0.0	0.0	0.0	0.0
1451096_at	Ndufs2	OXPHOS	0.0	0.0	0.0	0.0	1.4	1.5
1451312_at	Ndufs7	OXPHOS	0.0	0.0	0.0	0.0	1.3	1.4
1452184_at	Ndufb9	OXPHOS	0.0	0.0	0.0	0.0	1.2	1.2
1452692_a_at	Ndufv2	OXPHOS	0.0	0.0	0.0	0.0	1.3	1.3
1452790_x_at	Ndufa3	OXPHOS	0.0	0.0	0.0	0.0	1.4	1.4
1455036_s_at	Ndufc2	OXPHOS	0.0	0.0	0.0	0.0	0.0	1.2
1455283_x_at	Ndufs8	OXPHOS	0.0	0.0	0.0	0.0	1.3	1.4

1455749_x_at	Ndufa7	OXPHOS	0.0	0.0	0.0	1.5	1.5	1.2
1455806_x_at	2410011G03Rik	OXPHOS	0.0	0.0	0.0	0.0	0.0	0.0
1455911_x_at	Np15	OXPHOS	0.0	0.0	0.0	0.0	1.2	0.0
1456015_x_at	Ndufv1	OXPHOS	0.0	0.0	0.0	0.0	0.0	1.5
1415933_a_at	Cox5a	OXPHOS	0.0	0.0	0.0	0.0	1.5	1.3
1415970_at	Cox6c	OXPHOS	0.0	0.0	0.0	0.0	0.0	0.0
1416112_at	Cox8a	OXPHOS	0.0	0.0	0.0	0.0	0.0	0.0
1416565_at	Cox6b1	OXPHOS	0.0	0.0	0.0	0.0	1.3	0.0
1416604_at	Cyc1	OXPHOS	0.0	0.0	0.0	1.2	1.7	1.5
1416902_a_at	Cox5b	OXPHOS	0.0	0.0	0.0	0.0	1.3	1.3
1416970_a_at	Cox7a2	OXPHOS	0.0	0.0	0.0	0.0	0.0	0.0
1416971_at	Cox7a2	OXPHOS	0.0	0.0	0.0	0.0	0.0	1.3
1417417_a_at	Cox6a1	OXPHOS	0.0	0.0	0.0	0.0	0.0	0.0
1417418_s_at	Cox6a1	OXPHOS	0.0	0.0	0.0	0.0	0.0	1.3
1418709_at	Cox7a1	OXPHOS	0.0	0.0	0.0	0.0	2.1	4.6
1426693_x_at	Cox15	OXPHOS	0.0	0.0	0.0	0.0	0.0	1.3
1432264_x_at	Cox7a2l	OXPHOS	0.0	0.0	0.0	1.2	1.4	1.6
1434491_a_at	Cox6c	OXPHOS	0.0	0.0	0.0	0.0	0.0	0.0
1435613_x_at	Cox5b	OXPHOS	0.0	0.0	0.0	0.0	0.0	0.0
1436149_at	Cox5b	OXPHOS	0.0	0.0	0.0	0.0	1.4	0.0
1436409_at	Cox8a	OXPHOS	0.0	0.0	0.0	0.0	0.0	0.0
1436757_a_at	Cox6b1	OXPHOS	0.0	0.0	0.0	0.0	0.0	0.0
1437982_x_at	Cox15	OXPHOS	0.0	0.0	0.0	0.0	0.0	0.0
1439267_x_at	Cox5a	OXPHOS	0.0	0.0	0.0	0.0	0.0	2.0
1448112_at	Cox7c	OXPHOS	0.0	0.0	0.0	0.0	0.0	1.4
1448153_at	Cox5a	OXPHOS	0.0	0.0	0.0	1.3	1.8	0.0
1448222_x_at	Cox8a	OXPHOS	0.0	0.0	0.0	0.0	0.0	0.0
1448322_a_at	Cox4i1	OXPHOS	0.0	0.0	0.0	0.0	0.0	0.0
1449218_at	Cox8b	OXPHOS	0.0	0.0	0.0	0.0	1.4	1.5
1452146_a_at	Cox15	OXPHOS	0.0	0.0	0.0	0.0	0.0	1.3
1454716_x_at	Cox5b	OXPHOS	0.0	0.0	0.0	0.0	1.3	1.4
1455167_at	Cox8c	OXPHOS	0.0	0.0	0.0	0.0	0.0	0.0
1456588_x_at	Cox5b	OXPHOS	0.0	0.0	0.0	0.0	1.3	0.0
1416337_at	Uqcrb	OXPHOS	0.0	0.0	0.0	0.0	1.6	1.4
1424364_a_at	1110020P15Rik	OXPHOS	0.0	0.0	0.0	0.0	1.5	1.4
1427880_at	1500040F11Rik	OXPHOS	0.0	0.0	0.0	0.0	0.0	0.0
1428631_a_at	Uqcrc2	OXPHOS	0.0	0.0	0.0	0.0	1.5	1.3
1428782_a_at	Uqcrc1	OXPHOS	0.0	0.0	0.0	0.0	1.2	1.4
1430326_s_at	1500040F11Rik	OXPHOS	0.0	0.0	0.0	0.0	1.5	1.5
1435757_a_at	Uqcrc2	OXPHOS	0.0	0.0	0.0	0.0	1.4	0.0
1448292_at	Uqcr	OXPHOS	0.0	0.0	0.0	0.0	1.3	1.3
1450968_at	Uqcrfs1	OXPHOS	0.0	0.0	0.0	1.2	1.5	1.4
1452133_at	Uqcrh	OXPHOS	0.0	0.0	0.0	0.0	1.4	1.3
1452613_at	1500040F11Rik	OXPHOS	0.0	0.0	0.0	0.0	0.0	0.0
1455997_a_at	Uqcrb	OXPHOS	0.0	0.0	0.0	0.0	0.0	0.0
1422483_a_at	Cycs	OXPHOS	0.0	0.0	0.0	1.3	1.8	1.5
1422484_at	Cycs	OXPHOS	0.0	0.0	0.0	1.3	1.9	1.8
1418005_at	Sdhb	OXPHOS/TCA	0.0	0.0	0.0	0.0	1.4	1.6

1426688_at	Sdha	OXPHOS/TCA	0.0	0.0	0.0	0.0	0.0	1.3
1426689_s_at	Sdha	OXPHOS/TCA	0.0	0.0	0.0	0.0	0.0	1.3
1428235_at	Sdhd	OXPHOS/TCA	0.0	0.0	0.0	1.3	1.4	1.3
1435986_x_at	Sdhc	OXPHOS/TCA	0.0	0.0	0.0	0.0	2.2	0.0
1437489_x_at	Sdhd	OXPHOS/TCA	0.0	0.0	0.0	0.0	0.0	1.4
1448630_a_at	Sdhc	OXPHOS/TCA	0.0	0.0	0.0	0.0	1.3	1.3
1415891_at	Suclg1	TCA	0.0	0.0	0.0	0.0	1.4	1.3
1416090_at	Pdhb	TCA	0.0	0.0	0.0	0.0	1.3	0.0
1416383_a_at	Pcx	TCA	0.0	0.0	0.0	1.3	1.2	0.0
1416478_a_at	Mdh2	TCA	0.0	0.0	0.0	0.0	1.3	1.4
1416788_a_at	Idh3g	TCA	0.0	0.0	0.0	0.0	1.4	1.4
1416789_at	Idh3g	TCA	0.0	0.0	1.2	0.0	1.3	1.4
1417273_at	Pdk4	TCA	2.2	2.9	2.3	3.6	4.0	4.6
1418560_at	Pdha1	TCA	0.0	0.0	0.0	0.0	0.0	0.0
1418885_a_at	Idh3b	TCA	0.0	0.0	0.0	0.0	1.5	1.5
1418886_s_at	Idh3b	TCA	0.0	0.0	0.0	0.0	1.5	1.5
1422500_at	Idh3a	TCA	0.0	0.0	0.0	1.7	1.8	1.7
1422501_s_at	Idh3a	TCA	0.0	0.0	0.0	1.5	1.8	1.8
1422577_at	Cs	TCA	0.0	0.0	0.0	1.3	1.8	1.6
1422578_at	Cs	TCA	0.0	0.0	0.0	0.0	1.3	0.0
1423159_at	Dld	TCA	0.0	0.0	0.0	0.0	1.4	1.4
1423710_at	Dlst	TCA	0.0	0.0	0.0	0.0	1.4	1.5
1423747_a_at	Pdk1	TCA	0.0	0.0	0.0	0.0	1.9	1.5
1423748_at	Pdk1	TCA	0.0	0.0	0.0	0.0	2.3	1.8
1424828_a_at	Fh1	TCA	0.0	0.0	0.0	0.0	1.4	1.3
1425615_a_at	1810010O14Rik	TCA	0.0	0.0	0.0	0.0	0.0	0.0
1426264_at	Dlat	TCA	0.0	0.0	0.0	1.8	1.9	2.0
1426265_x_at	Dlat	TCA	0.0	0.0	0.0	1.6	2.1	2.1
1426410_at	Pdk3	TCA	0.0	0.0	0.0	0.0	0.0	0.0
1427441_a_at	Suclg2	TCA	0.0	0.0	0.0	0.0	-1.2	0.0
1431011_at	Dlst	TCA	0.0	0.0	0.0	2.3	0.0	0.0
1432016_a_at	Idh3a	TCA	0.0	0.0	0.0	1.5	1.5	1.6
1433984_a_at	Mdh2	TCA	0.0	0.0	0.0	0.0	1.3	0.0
1436934_s_at	Aco2	TCA	0.0	0.0	0.0	0.0	1.8	1.8
1448214_at	Pdhb	TCA	1.5	0.0	0.0	0.0	1.4	1.4
1448825_at	Pdk2	TCA	0.0	0.0	0.0	0.0	2.4	3.2
1449137_at	Pdha1	TCA	0.0	0.0	0.0	0.0	1.3	1.5
1450048_a_at	Idh2	TCA	0.0	0.0	0.0	0.0	0.0	0.0
1450667_a_at	Cs	TCA	0.0	0.0	0.0	0.0	1.5	1.5
1451002_at	Aco2	TCA	0.0	0.0	0.0	0.0	1.5	1.6
1451274_at	Ogdh	TCA	0.0	0.0	0.0	0.0	1.7	1.8
1452005_at	Dlat	TCA	0.0	0.0	1.4	1.7	1.6	1.7
1452206_at	Sucla2	TCA	0.0	0.0	0.0	0.0	1.6	1.3
1417956_at	Cidea	UCP	0.0	0.0	0.0	0.0	0.0	3.7
1418197_at	Ucp1	UCP	0.0	0.0	0.0	0.0	0.0	0.0
1420657_at	Ucp3	UCP	0.0	0.0	0.0	0.0	1.4	1.5
1420658_at	Ucp3	UCP	0.0	0.0	1.9	2.0	1.6	1.7
1448188_at	Ucp2	UCP	0.0	0.0	1.3	0.0	1.5	1.5

1416300_a_at	Slc25a3	ATP_Shuttle	0.0	0.0	0.0	0.0	0.0	0.0
1417434_at	Gpd2	ATP_Shuttle	0.0	0.0	0.0	0.0	0.0	0.0
1423108_at	Slc25a20	ATP_Shuttle	0.0	0.0	0.0	1.5	2.1	1.9
1423109_s_at	Slc25a20	ATP_Shuttle	0.0	0.0	0.0	1.6	2.4	1.7
1423772_x_at	Slc25a5	ATP_Shuttle	0.0	0.0	0.0	0.0	0.0	0.0
1424735_at	Slc25a25	ATP_Shuttle	0.0	0.0	0.0	0.0	0.0	0.0
1427483_at	Slc25a24	ATP_Shuttle	0.0	0.0	0.0	-2.0	-2.6	0.0
1428929_s_at	Slc25a26	ATP_Shuttle	0.0	0.0	0.0	0.0	0.0	1.5
1430542_a_at	Slc25a5	ATP_Shuttle	0.0	0.0	0.0	0.0	0.0	0.0
1434801_x_at	Slc25a5	ATP_Shuttle	0.0	0.0	0.0	1.4	0.0	0.0
1436874_x_at	Slc25a5	ATP_Shuttle	0.0	0.0	0.0	0.0	1.3	0.0
1438360_x_at	Slc25a5	ATP_Shuttle	0.0	0.0	0.0	0.0	0.0	0.0
1438546_x_at	Slc25a5	ATP_Shuttle	0.0	0.0	0.0	1.3	1.2	0.0
1438922_x_at	Slc25a5	ATP_Shuttle	0.0	0.0	0.0	0.0	1.7	0.0
1415990_at	Vdac2	VDAC	0.0	0.0	0.0	0.0	1.2	1.3
1415998_at	Vdac1	VDAC	0.0	0.0	0.0	0.0	1.5	1.3
1416175_a_at	Vdac3	VDAC	0.0	0.0	0.0	0.0	0.0	0.0
1436992_x_at	Vdac1	VDAC	0.0	0.0	0.0	0.0	1.2	1.3
1437192_x_at	Vdac1	VDAC	0.0	0.0	0.0	0.0	0.0	0.0
1437947_x_at	Vdac1	VDAC	0.0	0.0	0.0	0.0	2.0	0.0
**Probe sets in Figure 3A**								
1417066_at	Cabc1	MitHSP	0.0	0.0	0.0	-1.3	0.0	0.0
1417101_at	Hspa2	MitHSP	0.0	0.0	0.0	0.0	-1.6	0.0
1417320_at	Grpel1	MitHSP	0.0	0.0	0.0	1.3	1.6	1.4
1418503_at	Hspa9a	MitHSP	0.0	0.0	0.0	0.0	0.0	1.5
1418504_at	Hspa9a	MitHSP	0.0	0.0	0.0	0.0	1.3	0.0
1420629_a_at	Dnaja3	MitHSP	0.0	0.0	0.0	0.0	0.0	1.7
1422579_at	Hspe1	MitHSP	0.0	0.0	0.0	1.3	1.2	1.3
1426351_at	Hspd1	MitHSP	0.0	0.0	0.0	0.0	0.0	1.3
1431274_a_at	Hspa9a	MitHSP	0.0	0.0	0.0	0.0	0.0	0.0
1449935_a_at	Dnaja3	MitHSP	0.0	0.0	0.0	1.3	1.3	1.6
1450668_s_at	Hspe1	MitHSP	0.0	0.0	0.0	0.0	0.0	0.0
1452262_at	Grpel2	MitHSP	0.0	0.0	0.0	0.0	0.0	0.0
1415681_at	Mrpl43	MitRibosomal protein	0.0	0.0	0.0	0.0	0.0	0.0
1415690_at	Mrpl27	MitRibosomal protein	0.0	0.0	0.0	0.0	0.0	0.0
1415762_x_at	Mrpl52	MitRibosomal protein	0.0	0.0	0.0	0.0	0.0	0.0
1416284_at	Mrpl28	MitRibosomal protein	0.0	0.0	0.0	0.0	0.0	0.0
1416349_at	Mrpl34	MitRibosomal protein	0.0	0.0	0.0	1.3	1.3	1.6
1416510_at	Mrpl4	MitRibosomal protein	0.0	0.0	0.0	0.0	0.0	1.2
1416595_at	Mrps22	MitRibosomal protein	0.0	0.0	0.0	0.0	0.0	0.0
1416877_a_at	Mrpl51	MitRibosomal protein	0.0	0.0	0.0	0.0	1.7	1.2
1416879_at	Mrpl51	MitRibosomal protein	0.0	0.0	0.0	1.6	0.0	0.0
1416984_at	Mrps18a	MitRibosomal protein	0.0	0.0	0.0	0.0	0.0	0.0
1417966_at	Mrpl39	MitRibosomal protein	0.0	0.0	0.0	0.0	0.0	0.0
1418112_at	Mrpl10	MitRibosomal protein	0.0	0.0	0.0	0.0	0.0	0.0
1418137_at	Mrp63	MitRibosomal protein	0.0	0.0	0.0	0.0	0.0	0.0
1418716_at	Mrps25	MitRibosomal protein	0.0	0.0	0.0	0.0	0.0	0.0
1419362_at	Mrpl35	MitRibosomal protein	0.0	0.0	0.0	0.0	0.0	0.0

1419363_a_at	Mrpl35	MitRibosomal protein	0.0	0.0	0.0	0.0	0.0	0.0
1420488_at	Mrps14	MitRibosomal protein	0.0	0.0	0.0	0.0	0.0	0.0
1420489_at	Mrps14	MitRibosomal protein	0.0	0.0	0.0	0.0	0.0	0.0
1420845_at	Mrps2	MitRibosomal protein	0.0	0.0	0.0	0.0	0.0	1.6
1420846_at	Mrps2	MitRibosomal protein	0.0	0.0	0.0	0.0	1.2	1.4
1421874_a_at	Mrps23	MitRibosomal protein	0.0	0.0	0.0	1.3	1.4	1.3
1421875_a_at	Mrps23	MitRibosomal protein	0.0	0.0	0.0	0.0	0.0	1.4
1421914_s_at	Mrpl19	MitRibosomal protein	0.0	0.0	0.0	0.0	0.0	1.4
1421971_a_at	Mrps34	MitRibosomal protein	0.0	0.0	1.2	1.2	0.0	1.2
1422451_at	Mrps21	MitRibosomal protein	0.0	0.0	0.0	0.0	1.3	0.0
1422819_at	Mrpl36	MitRibosomal protein	0.0	0.0	0.0	0.0	0.0	0.0
1423219_a_at	Mrpl49	MitRibosomal protein	0.0	0.0	0.0	0.0	0.0	0.0
1423242_at	Mrps36	MitRibosomal protein	0.0	0.0	0.0	0.0	1.3	0.0
1423492_at	Mrpl45	MitRibosomal protein	0.0	0.0	0.0	0.0	1.6	1.3
1423764_s_at	Mrpl37	MitRibosomal protein	0.0	0.0	0.0	0.0	0.0	1.2
1423857_at	Mrpl30	MitRibosomal protein	0.0	0.0	0.0	0.0	1.2	1.3
1424164_at	Mrpl50	MitRibosomal protein	0.0	0.0	0.0	0.0	0.0	0.0
1424204_at	Mrpl13	MitRibosomal protein	0.0	0.0	0.0	0.0	0.0	0.0
1424372_at	Mrpl32	MitRibosomal protein	0.0	0.0	0.0	0.0	0.0	0.0
1424440_at	Mrps6	MitRibosomal protein	0.0	0.0	1.2	1.2	0.0	0.0
1425189_a_at	Mrpl15	MitRibosomal protein	0.0	0.0	0.0	1.3	1.5	1.3
1426651_at	Mrpl44	MitRibosomal protein	0.0	0.0	0.0	0.0	0.0	0.0
1427158_at	Mrps30	MitRibosomal protein	0.0	0.0	0.0	0.0	0.0	0.0
1427173_a_at	Mrps33	MitRibosomal protein	0.0	0.0	0.0	0.0	0.0	0.0
1427297_at	Mrpl9	MitRibosomal protein	0.0	0.0	0.0	0.0	1.4	1.4
1427901_at	Mrps18c	MitRibosomal protein	0.0	0.0	0.0	0.0	0.0	0.0
1428302_at	Mrpl48	MitRibosomal protein	0.0	0.0	0.0	0.0	1.6	0.0
1428709_a_at	Mrpl24	MitRibosomal protein	0.0	0.0	0.0	0.0	0.0	0.0
1429054_at	Mrpl47	MitRibosomal protein	0.0	0.0	0.0	1.4	1.3	1.5
1429453_a_at	Mrpl55	MitRibosomal protein	0.0	0.0	0.0	1.3	1.4	1.4
1430976_a_at	Mrpl9	MitRibosomal protein	0.0	0.0	0.0	0.0	0.0	0.0
1433878_at	Mrps26	MitRibosomal protein	0.0	0.0	0.0	0.0	0.0	0.0
1434971_x_at	Mrpl15	MitRibosomal protein	0.0	0.0	0.0	0.0	1.4	0.0
1435232_x_at	Mrpl15	MitRibosomal protein	0.0	0.0	0.0	1.3	1.4	1.4
1435843_x_at	Mrps9	MitRibosomal protein	0.0	0.0	0.0	0.0	0.0	0.0
1435966_x_at	Mrpl13	MitRibosomal protein	0.0	0.0	0.0	0.0	0.0	0.0
1435995_at	Mrpl22	MitRibosomal protein	0.0	0.0	0.0	0.0	0.0	0.0
1437131_x_at	Mrpl11	MitRibosomal protein	0.0	0.0	0.0	0.0	0.0	0.0
1437391_x_at	Mrpl44	MitRibosomal protein	0.0	0.0	0.0	0.0	0.0	0.0
1437622_x_at	Mrpl28	MitRibosomal protein	0.0	0.0	0.0	0.0	0.0	0.0
1437839_x_at	Mrpl11	MitRibosomal protein	0.0	0.0	0.0	0.0	0.0	0.0
1437997_x_at	Mrpl48	MitRibosomal protein	0.0	0.0	0.0	0.0	0.0	1.3
1438014_at	Mrpl34	MitRibosomal protein	0.0	0.0	0.0	0.0	0.0	1.8
1438563_s_at	Mrps24	MitRibosomal protein	0.0	0.0	0.0	0.0	0.0	0.0
1439210_at	Mrps9	MitRibosomal protein	0.0	0.0	0.0	0.0	0.0	0.0
1447961_s_at	Mrpl38	MitRibosomal protein	0.0	0.0	0.0	0.0	0.0	0.0
1448373_at	Mrpl18	MitRibosomal protein	0.0	0.0	0.0	1.4	1.5	1.6
1448488_at	Mrps5	MitRibosomal protein	0.0	0.0	1.2	0.0	0.0	0.0

1448699_at	Mrpl54	MitRibosomal protein	0.0	0.0	0.0	0.0	0.0	0.0
1448799_s_at	Mrps12	MitRibosomal protein	0.0	0.0	0.0	0.0	0.0	0.0
1448811_at	Mrpl2	MitRibosomal protein	0.0	0.0	0.0	0.0	0.0	0.0
1448849_at	Mrpl40	MitRibosomal protein	0.0	0.0	0.0	0.0	0.0	0.0
1448869_a_at	Mrps16	MitRibosomal protein	0.0	0.0	0.0	0.0	1.3	1.4
1448909_a_at	Mrpl39	MitRibosomal protein	0.0	0.0	0.0	0.0	0.0	0.0
1448921_a_at	Mrps9	MitRibosomal protein	0.0	0.0	0.0	0.0	0.0	1.4
1449004_at	Mrpl46	MitRibosomal protein	0.0	0.0	0.0	1.3	1.2	1.3
1449194_at	Mrps25	MitRibosomal protein	0.0	0.0	0.0	0.0	0.0	0.0
1449294_at	Mrps15	MitRibosomal protein	0.0	0.0	0.0	0.0	0.0	0.0
1450405_at	Mrpl19	MitRibosomal protein	0.0	0.0	0.0	1.6	1.5	1.4
1450866_a_at	Mrpl17	MitRibosomal protein	0.0	0.0	0.0	0.0	0.0	0.0
1450867_at	Mrpl17	MitRibosomal protein	0.0	0.0	0.0	-1.7	0.0	0.0
1450880_at	Mrpl16	MitRibosomal protein	0.0	0.0	0.0	0.0	0.0	1.3
1450948_a_at	Mrpl1	MitRibosomal protein	0.0	0.0	0.0	0.0	0.0	0.0
1451164_a_at	Mrps18b	MitRibosomal protein	0.0	0.0	0.0	1.2	1.4	1.4
1451178_at	Mrpl53	MitRibosomal protein	0.0	0.0	0.0	0.0	0.0	0.0
1451266_at	Mrpl50	MitRibosomal protein	0.0	0.0	0.0	0.0	0.0	1.3
1451307_at	Mrpl14	MitRibosomal protein	0.0	0.0	0.0	0.0	0.0	0.0
1452048_at	Mrpl12	MitRibosomal protein	1.2	0.0	0.0	1.7	1.6	1.5
1452144_a_at	Mrpl44	MitRibosomal protein	0.0	0.0	0.0	0.0	0.0	0.0
1452585_at	Mrps28	MitRibosomal protein	0.0	0.0	0.0	0.0	0.0	0.0
1453571_at	Mrpl19	MitRibosomal protein	0.0	0.0	1.3	0.0	0.0	0.0
1453725_a_at	Mrps7	MitRibosomal protein	0.0	0.0	0.0	0.0	0.0	0.0
1453728_a_at	Mrps17	MitRibosomal protein	0.0	0.0	0.0	1.3	0.0	0.0
1453954_a_at	Mrps5	MitRibosomal protein	0.0	0.0	0.0	0.0	0.0	1.4
1456109_a_at	Mrps15	MitRibosomal protein	0.0	0.0	0.0	0.0	0.0	0.0
1456313_x_at	Mrpl28	MitRibosomal protein	0.0	0.0	0.0	0.0	0.0	0.0
1460195_at	Mrps11	MitRibosomal protein	0.0	-1.3	0.0	0.0	0.0	0.0
1460225_at	Mrp63	MitRibosomal protein	0.0	0.0	0.0	-1.3	0.0	0.0
1460354_a_at	Mrpl13	MitRibosomal protein	0.0	0.0	0.0	0.0	0.0	0.0
1460701_a_at	Mrpl52	MitRibosomal protein	0.0	0.0	0.0	0.0	0.0	0.0
1416345_at	Timm8a	TIMMS & TOMMS	0.0	0.0	1.6	2.1	1.4	1.4
1416346_at	Timm8a	TIMMS & TOMMS	0.0	0.0	0.0	1.7	1.5	1.6
1416485_at	Timm23	TIMMS & TOMMS	0.0	0.0	0.0	1.4	1.3	1.2
1417192_at	Tomm70a	TIMMS & TOMMS	0.0	0.0	0.0	1.6	1.4	0.0
1417499_at	Timm13a	TIMMS & TOMMS	0.0	0.0	0.0	1.4	0.0	0.0
1417670_at	Timm44	TIMMS & TOMMS	0.0	0.0	0.0	1.3	1.5	1.5
1417916_a_at	Fxc1	TIMMS & TOMMS	0.0	0.0	0.0	0.0	0.0	0.0
1423079_a_at	Tomm20	TIMMS & TOMMS	0.0	0.0	0.0	0.0	1.3	0.0
1423080_at	Tomm20	TIMMS & TOMMS	0.0	0.0	0.0	1.2	0.0	0.0
1423081_a_at	Tomm20	TIMMS & TOMMS	0.0	0.0	0.0	0.0	0.0	0.0
1426118_a_at	Tomm40	TIMMS & TOMMS	0.0	0.0	0.0	1.2	1.3	1.4
1426256_at	Timm17a	TIMMS & TOMMS	0.0	0.0	0.0	1.2	1.4	0.0
1426675_at	Tomm70a	TIMMS & TOMMS	0.0	0.0	0.0	0.0	0.0	0.0
1426676_s_at	Tomm70a	TIMMS & TOMMS	0.0	0.0	1.3	1.4	0.0	1.6
1428214_at	Tomm7	TIMMS & TOMMS	0.0	0.0	0.0	0.0	0.0	0.0
1428215_x_at	Tomm7	TIMMS & TOMMS	0.0	0.0	0.0	0.0	0.0	0.0

1428216_s_at	Tomm7	TIMMS & TOMMS	0.0	0.0	0.0	0.0	0.0	0.0
1431665_a_at	Timm8b	TIMMS & TOMMS	0.0	0.0	0.0	0.0	0.0	1.5
1434732_x_at	Tomm7	TIMMS & TOMMS	0.0	0.0	0.0	0.0	0.0	0.0
1435534_a_at	Tomm20	TIMMS & TOMMS	0.0	0.0	0.0	0.0	0.0	0.0
1438656_x_at	Timm17b	TIMMS & TOMMS	0.0	0.0	0.0	0.0	0.0	-1.4
1439371_x_at	Timm44	TIMMS & TOMMS	0.0	0.0	0.0	0.0	0.0	0.0
1448345_at	Tomm34	TIMMS & TOMMS	0.0	0.0	0.0	0.0	0.0	0.0
1448517_at	Timm22	TIMMS & TOMMS	0.0	0.0	1.3	1.4	1.4	0.0
1448801_a_at	Timm44	TIMMS & TOMMS	0.0	0.0	0.0	0.0	1.3	1.5
1449886_a_at	Timm10	TIMMS & TOMMS	0.0	0.0	0.0	1.9	1.6	0.0
1455211_a_at	Timm9	TIMMS & TOMMS	0.0	0.0	0.0	0.0	1.3	1.3
1455357_x_at	Tomm20	TIMMS & TOMMS	0.0	0.0	0.0	1.6	0.0	0.0
1455456_a_at	Timm50	TIMMS & TOMMS	0.0	0.0	0.0	0.0	0.0	0.0
1460685_at	Timm17b	TIMMS & TOMMS	0.0	0.0	0.0	0.0	0.0	0.0
**Probe sets in Figure 3B**								
1429533_at	Immt	Structure	0.0	0.0	0.0	0.0	0.0	1.4
1429534_a_at	Immt	Structure	0.0	0.0	0.0	0.0	1.2	1.3
1433470_a_at	Immt	Structure	0.0	0.0	0.0	0.0	1.4	1.4
1418768_at	Opa1	Structure	0.0	0.0	0.0	0.0	0.0	0.0
1449214_a_at	Opa1	Structure	0.0	0.0	0.0	0.0	1.5	1.6
1451541_at	Bcs1l	Structure	0.0	0.0	0.0	0.0	1.4	1.4
1450561_a_at	Surf1	Structure	0.0	0.0	0.0	0.0	-1.3	0.0
**Probe sets in Figure 3C**								
1460652_at	Esrra	Transcription factor	0.0	0.0	0.0	1.6	1.4	1.5
1450664_at	Gabpa	Transcription factor	0.0	0.0	0.0	0.0	0.0	0.0
1450665_at	Gabpa	Transcription factor	0.0	0.0	0.0	0.0	0.0	0.0
1436232_a_at	Gabpb1	Transcription factor	0.0	0.0	0.0	0.0	0.0	0.0
1419761_a_at	Gabpb1	Transcription factor	0.0	0.0	0.0	0.0	0.0	0.0
1450444_a_at	Nr1h3	Transcription factor	0.0	0.0	0.0	0.0	0.0	0.0
1424787_a_at	Nrf1	Transcription factor	0.0	0.0	0.0	0.0	0.0	0.0
1434627_at	Nrf1	Transcription factor	0.0	0.0	0.0	0.0	0.0	0.0
1418469_at	Nrip1	Transcription factor	0.0	2.2	0.0	0.0	0.0	0.0
1449089_at	Nrip1	Transcription factor	0.0	2.2	0.0	1.7	0.0	0.0
1449051_at	Ppara	Transcription factor	0.0	0.0	0.0	8.4	9.9	9.0
1460336_at	Ppargc1a	Transcription factor	0.0	0.0	0.0	0.0	2.3	0.0
1449945_at	Ppargc1b	Transcription factor	0.0	0.0	3.3	3.2	2.1	1.9
1426381_at	Pprc1	Transcription factor	0.0	0.0	0.0	0.0	0.0	0.0
1448224_at	Tfam	Transcription factor	0.0	0.0	0.0	0.0	0.0	0.0
1449749_s_at	Tfb1m	Transcription factor	0.0	0.0	0.0	0.0	1.2	0.0
1423441_at	Tfb2m	Transcription factor	1.3	0.0	0.0	0.0	0.0	0.0
**Probe sets in Supplemental Figure 3**								
1417867_at	Adn		0.0	0.0	0.0	0.0	-1.6	-1.4
1423828_at	Fasn		0.0	0.0	0.0	0.0	0.0	0.0
1434773_a_at	Slc2a1		0.0	0.0	0.0	0.0	0.0	0.0
1426600_at	Slc2a1		0.0	0.0	0.0	0.0	0.0	0.0
1426599_a_at	Slc2a1		0.0	0.0	0.0	0.0	0.0	0.0
**Probe sets mentioned in Discussion**								
1422820_at	Lipe		0.0	0.0	1.3	1.3	0.0	0.0

1428143_a_at	Pnpla2		0.0	0.0	0.0	0.0	0.0	0.0
1428591_at	Pnpla2		0.0	0.0	1.5	1.5	0.0	1.7
1427367_at	Pnpla2		0.0	0.0	0.0	0.0	0.0	0.0

Supplemental Table 5. Confirmation of the microarray data using QRT-PCR. The numerical data are expressed as mean –ΔΔCt, equivalent to log2-transformed fold changes (RSG over DMSO). N = 3 per time point in each cell type. *p < 0.05.

3T3 L1 Adipocytes

Gene Name	1hr	2hr	4hr	7hr	24hr	48hr
Hadha	-0.27	0.41	0.32	0.5 *	1.35 *	1.97 *
Cidea	-0.16	-0.12	0.34	0.69	2.07 *	3.82 *
Cpt1b	-0.21	0.3	0.52	0.63	2.67 *	3.74 *
Cs	-0.15	0.2	0.15	0.32	0.97 *	1.18 *
Cycs	-0.09	0.57 *	0.77 *	1.4 *	2.23 *	2.57 *
Idh3a	-0.12	0.46	0.61 *	0.93 *	2.05 *	2.33 *
Opa1	-0.22	0.29	0.31	0.24	0.73 *	1.08 *
Ppargc1b	-0.02	1.05 *	0.98 *	1.31 *	1.78 *	1.62 *

C3H10T1/2 Adipocytes

Gene Name	1hr	2hr	4hr	7hr	24hr	48hr
Hadha	0.06	0.11	0.22	-0.02	1.21 *	1.28 *
Cidea	0.06	0.11	0.22	-0.06	1.5 *	1.91 *
Cpt1b	0.02	0.01	0.28	0.6 *	3.34 *	3.41 *
Cs	0.04	-0.08	0.14	0.11	0.73 *	0.62 *
Cycs	0.04	0.1	0.28	0.23	1.51 *	1.34 *
Idh3a	0.07	-0.06	0.63 *	1.09 *	1.53 *	1.3 *
Opa1	0.01	0.11	0.35	-0.31	0.87 *	0.52
Ppargc1b	0.22	1.31 *	1.88 *	1.71 *	1.29 *	1.31 *

Supplemental Table 6. Presence of PPAR-responsive element consensus regions on the promoters of nucleus-encoded mitochondrial genes. The gene promoter sequences were acquired from PromoSer database (http://biowulf.bu.edu/zlab/PromoSer/). Each promoter contains 2000 bases upstream and 100 bases downstream relative to the 5' transcription start site (TSS). The PPAR-responsive elements on gene promoters are identified through MATCH program using transcription factor binding matrix of PPAR_DR1_Q2 and PPARG_03 in TRANSFAC Professional database (V8.1, http://www.biobase-international.com). Only genes with promoters containing sequences with an exact match (score = 1) of the core elements and a score of > 0.75 for the matrix match are listed.

Gene Symbol	Reference Sequence Number	Category	Number of matches	Positions Relative to TSS	Regulation in 3T3-L1	Regulation in C3H/10T1/2
Acad9	NM_172678	FAO	1	-1385,	UP	NC
Acadl	NM_007381	FAO	1	-1851,	UP	UP
Acadm	NM_007382	FAO	1	-198,	UP	UP
Acat2	NM_009338	FAO	1	-558,	UP	UP
Acox1	NM_015729	FAO	4	52, -297, -440, -1478,	UP	UP
Acsl5	NM_027976	FAO	5	-43, -119, -221, -1115, -1853,	Down	Down
Cpt1b	NM_009948	FAO	2	-246, -1454,	UP	UP
Decr1	NM_026172	FAO	1	-1227,	UP	UP
Ech1	NM_016772	FAO	3	-134, -559, -1123,	UP	UP
Echdc3	NM_024208	FAO	1	-1388,	NC	NC
Echs1	NM_053119	FAO	1	-192,	UP	NC
Gcdh	NM_001044744	FAO	1	-1516,	NC	NC
Hadhb	NM_145558	FAO	1	-306,	UP	UP
Hadhsc	NM_008212	FAO	1	45,	UP	UP
Hibch	NM_146108	FAO	1	-150,	UP	NC

Hsd17b4	NM_008292	FAO	2	-1060, -1360,	UP	UP
MGC29978	NM_146230	FAO	3	-580, -825, -954,	UP	UP
Psap	NM_011179	FAO	1	-1049,	NC	NC
Etfb	NM_026695	OXPHOS/FAO	1	-141,	UP	UP
0610006O14Rik	NM_133764	OXPHOS	1	-1678,	Down	Down
1500032D16Rik	NM_030087	OXPHOS	1	-448,	UP	UP
Atp5b	NM_016774	OXPHOS	2	-601, -622,	UP	NC
Atp5c1	NM_020615	OXPHOS	1	-1236,	UP	UP
Atp5d	NM_025313	OXPHOS	1	-674,	UP	UP
Atp5f1	NM_009725	OXPHOS	2	-389, -471,	UP	UP
Atp5g3	NM_175015	OXPHOS	1	-1099,	UP	NC
Atp5j2	NM_020582	OXPHOS	1	-1869,	UP	UP
Atp5l	NM_013795	OXPHOS	2	-385, -1491,	UP	UP
Atp5o	NM_138597	OXPHOS	1	-924,	UP	UP
Atp6v1a1	NM_007508	OXPHOS	1	-31,	NC	NC
Cox15	NM_144874	OXPHOS	1	-1786,	UP	UP
Cox5a	NM_007747	OXPHOS	1	-451,	UP	UP
Cox5b	NM_009942	OXPHOS	3	-154, -489, -1087,	UP	UP
Cox6a1	NM_007748	OXPHOS	1	-579,	UP	UP
Cox7a1	NM_009944	OXPHOS	1	-825,	UP	UP
Cox7a2l	NM_009187	OXPHOS	1	-1467,	UP	UP
Cox8a	NM_007750	OXPHOS	2	-1082, -1987,	UP	NC
Cyc1	NM_025567	OXPHOS	1	-629,	UP	UP
Cycs	NM_007808	OXPHOS	2	-360, -434,	UP	UP
Ndufa1	NM_019443	OXPHOS	1	-1111,	UP	UP
Ndufa10	NM_024197	OXPHOS	2	-135, -1078,	UP	UP
Ndufa2	NM_010885	OXPHOS	1	71,	NC	UP
Ndufa8	NM_026703	OXPHOS	1	-925,	UP	UP
Ndufa9	NM_025358	OXPHOS	2	-380, -1479,	UP	UP

Ndufab1	NM_028177	OXPHOS	1	-1966,	UP	UP
Ndufb10	NM_026684	OXPHOS	2	-894, -1166,	UP	UP
Ndufb2	NM_026612	OXPHOS	1	-117,	UP	NC
Ndufb5	NM_025316	OXPHOS	2	-922, -1310,	UP	UP
Ndufc2	NM_024220	OXPHOS	1	-31,	UP	UP
Ndufs1	NM_145518	OXPHOS	3	69, 2, -761,	UP	UP
Ndufs2	NM_153064	OXPHOS	1	-470,	UP	UP
Ndufs7	NM_029272	OXPHOS	1	-1173,	UP	UP
Ndufs8	NM_144870	OXPHOS	3	-660, -854, -986,	UP	UP
Ndufv1	NM_133666	OXPHOS	1	-1200,	UP	UP
Ndufv2	NM_028388	OXPHOS	1	-1934,	UP	UP
Np15	NM_019435	OXPHOS	1	-961,	UP	UP
Uqcr	NM_025650	OXPHOS	3	-577, -1208, -1626,	UP	UP
Uqcrc2	NM_025899	OXPHOS	1	-25,	UP	UP
Uqcrfs1	NM_025710	OXPHOS	3	-1616, -1728, -1954,	UP	UP
Uqcrh	NM_025641	OXPHOS	1	-1750,	UP	UP
Sdhb	NM_023374	OXPHOS/TCA	3	-24, -676, -1841,	UP	UP
Aco2	NM_080633	TCA	3	-103, -457, -1771,	UP	UP
Cs	NM_026444	TCA	2	-203, -1008,	UP	UP
Dlat	NM_145614	TCA	1	-185,	UP	UP
Dld	NM_007861	TCA	2	-1549, -1736,	UP	UP
Fh1	NM_010209	TCA	1	-1238,	UP	UP
Idh2	NM_173011	TCA	2	-736, -1367,	NC	NC
Idh3a	NM_029573	TCA	1	-1063,	UP	UP
Idh3b	NM_130884	TCA	1	-1042,	UP	UP
Pck2	NM_028994	TCA	2	-1764, -1970,	Down	NC
Pcx	NM_008797	TCA	2	-1660, -1666,	UP	UP
Pdha1	NM_008810	TCA	2	-282, -410,	UP	UP
Pdhb	NM_024221	TCA	1	-628,	UP	UP

Pdk1	NM_172665	TCA	1	-1617,	UP	UP
Pdk3	NM_145630	TCA	1	-664,	Down	NC
Pdk4	NM_013743	TCA	1	-1279,	UP	UP
Sucla2	NM_011506	TCA	1	-205,	UP	UP
Suclg1	NM_019879	TCA	2	-169, -1485,	UP	UP
Suclg2	NM_011507	TCA	1	-1604,	NC	Down
Ucp1	NM_009463	UCP	1	58,	NC	NC
Ucp3	NM_009464	UCP	1	-49,	UP	UP
Gpd2	NM_010274	ATP_Shuttle	2	-496, -1073,	NC	NC
Slc25a20	NM_020520	ATP_Shuttle	2	88, -764,	UP	UP
Slc25a25	NM_146118	ATP_Shuttle	2	-1569, -1694,	UP	NC
Slc25a3	NM_133668	ATP_Shuttle	2	-1051, -1928,	UP	NC
Slc25a5	NM_007451	ATP_Shuttle	1	-1030,	UP	UP
Vdac2	NM_011695	VDAC	1	-599,	UP	UP
Vdac3	NM_011696	VDAC	1	-185,	NC	NC
Dnaja3	NM_023646	MitHSP	1	-1352,	UP	UP
Grpel1	NM_024478	MitHSP	1	-199,	UP	UP
Grpel2	NM_021296	MitHSP	1	-1753,	UP	NC
Hspa9a	NM_010481	MitHSP	2	-237, -447,	UP	UP
Hspd1	NM_010477	MitHSP	2	-708, -1184,	UP	UP
Hspe1	NM_008303	MitHSP	3	-41, -1339, -1373,	UP	UP
Mrpl13	NM_026759	MitRibosomal protein	2	-84, -1970,	Down	NC
Mrpl14	NM_026732	MitRibosomal protein	3	-596, -928, -1217,	NC	NC
Mrpl15	NM_025300	MitRibosomal protein	2	-1292, -1862,	UP	UP
Mrpl16	NM_025606	MitRibosomal protein	2	-101, -1640,	NC	UP
Mrpl17	NM_025301	MitRibosomal protein	2	-435, -444,	Down	NC
Mrpl18	NM_026310	MitRibosomal protein	1	-242,	UP	UP
Mrpl2	NM_025302	MitRibosomal protein	1	-1309,	Down	NC
Mrpl22	NM_175001	MitRibosomal protein	1	-1765,	Down	NC

Mrpl24	NM_026591	MitRibosomal protein	3	-1279, -1731, -1848,	Down	NC
Mrpl28	NM_024227	MitRibosomal protein	1	-891,	NC	NC
Mrpl30	NM_027098	MitRibosomal protein	1	-1238,	NC	UP
Mrpl36	NM_053163	MitRibosomal protein	1	-17,	NC	NC
Mrpl37	NM_025500	MitRibosomal protein	1	-643,	NC	UP
Mrpl38	NM_024177	MitRibosomal protein	1	-745,	NC	NC
Mrpl40	NM_010922	MitRibosomal protein	1	-1263,	Down	NC
Mrpl45	NM_025927	MitRibosomal protein	1	-357,	UP	UP
Mrpl46	NM_023331	MitRibosomal protein	1	-406,	UP	UP
Mrpl49	NM_026246	MitRibosomal protein	4	-437, -867, -1426, -1553,	UP	NC
Mrpl50	NM_178603	MitRibosomal protein	1	-57,	NC	UP
Mrpl53	NM_026744	MitRibosomal protein	1	-1799,	UP	NC
Mrpl55	NM_026035	MitRibosomal protein	2	-1794, -1954,	UP	UP
Mrpl9	NM_030116	MitRibosomal protein	1	-1950,	UP	UP
Mrps11	NM_026498	MitRibosomal protein	1	-1624,	NC	Down
Mrps12	NM_011885	MitRibosomal protein	2	-1778, -1936,	NC	NC
Mrps15	NM_025544	MitRibosomal protein	1	-1179,	Down	NC
Mrps16	NM_025440	MitRibosomal protein	1	-1859,	UP	UP
Mrps17	NM_025450	MitRibosomal protein	1	-1364,	NC	UP
Mrps18a	NM_026768	MitRibosomal protein	1	-880,	UP	NC
Mrps18c	NM_026826	MitRibosomal protein	1	-1452,	NC	NC
Mrps2	NM_080452	MitRibosomal protein	2	-168, -1535,	UP	UP
Mrps22	NM_025485	MitRibosomal protein	3	13, -16, -754,	NC	NC
Mrps24	NM_026080	MitRibosomal protein	1	-1676,	NC	NC
Mrps28	NM_025434	MitRibosomal protein	2	47, -133,	UP	NC
Mrps33	NM_010270	MitRibosomal protein	2	-55, -116,	NC	NC
Mrps34	NM_023260	MitRibosomal protein	1	-1929,	UP	UP
Mrps36	NM_025369	MitRibosomal protein	1	-362,	NC	UP
Mrps5	NM_029963	MitRibosomal protein	1	-732,	UP	UP

Timm13a	NM_013899	TIMMS & TOMMS	3	-685, -790, -957,	UP	UP
Timm50	NM_025616	TIMMS & TOMMS	2	-408, -1027,	NC	NC
Timm8a	NM_013898	TIMMS & TOMMS	1	-892,	UP	UP
Timm8b	NM_013897	TIMMS & TOMMS	2	-99, -1676,	NC	UP
Tomm20	NM_024214	TIMMS & TOMMS	1	-1743,	UP	UP
Tomm70a	NM_138599	TIMMS & TOMMS	3	11, -176, -1896,	UP	UP
Immt	NM_029673	Structure	1	-1904,	UP	UP
Esrra	NM_007953	Transcription factor	2	-853, -1891,	UP	UP
Gabpa	NM_008065	Transcription factor	3	-207, -275, -713,	UP	UP
Nrf1	NM_010938	Transcription factor	1	-983,	UP	UP
Ppara	NM_001113418	Transcription factor	1	-754,	UP	UP
Ppargc1b	NM_133249	Transcription factor	2	-494, -1986,	UP	UP
